# hnRNP A1 dysfunction alters RNA splicing and drives neurodegeneration in multiple sclerosis (MS)

**DOI:** 10.1038/s41467-023-44658-1

**Published:** 2024-01-08

**Authors:** Hannah E. Salapa, Patricia A. Thibault, Cole D. Libner, Yulian Ding, Joseph-Patrick W. E. Clarke, Connor Denomy, Catherine Hutchinson, Hashim M. Abidullah, S. Austin Hammond, Landon Pastushok, Frederick S. Vizeacoumar, Michael C. Levin

**Affiliations:** 1 Office of the Saskatchewan Multiple Sclerosis Clinical Research Chair, University of Saskatchewan, Saskatoon, SK S7K 0M7 Canada; 2 Cameco MS Neuroscience Research Centre, College of Medicine, University of Saskatchewan, Saskatoon, SK S7K 0M7 Canada; 3 Neurology Division, Department of Medicine, University of Saskatchewan, Saskatoon, SK S7N 0X8 Canada; 4 Department of Health Sciences, College of Medicine, University of Saskatchewan, Saskatoon, SK S7N 5E5 Canada; 5 Division of Oncology, College of Medicine, University of Saskatchewan, Saskatoon, SK S7N 5E5 Canada; 6 Division of Biomedical Engineering, College of Medicine, University of Saskatchewan, Saskatoon, SK S7N 5A9 Canada; 7 Department of Anatomy, Physiology and Pharmacology, College of Medicine, University of Saskatchewan, Saskatoon, SK S7N 5E5 Canada; 8 Next-Generation Sequencing Facility, University of Saskatchewan, Saskatoon, SK S7N 5E5 Canada; 9 Advanced Diagnostics Research Laboratory, Department of Pathology and Lab Medicine, College of Medicine, University of Saskatchewan, Saskatoon, SK S7N 5E5 Canada; 10 Department of Pathology and Laboratory Medicine, University of Saskatchewan, Saskatoon, SK S7N 5E5 Canada

**Keywords:** Multiple sclerosis, RNA metabolism

## Abstract

Neurodegeneration is the primary driver of disease progression in multiple sclerosis (MS) resulting in permanent disability, creating an urgent need to discover its underlying mechanisms. Herein, we establish that dysfunction of the RNA binding protein heterogeneous nuclear ribonucleoprotein A1 (hnRNP A1) results in differential of binding to RNA targets causing alternative RNA splicing, which contributes to neurodegeneration in MS and its models. Using RNAseq of MS brains, we discovered differential expression and aberrant splicing of hnRNP A1 target RNAs involved in neuronal function and RNA homeostasis. We confirmed this in vivo in experimental autoimmune encephalomyelitis employing CLIPseq specific for hnRNP A1, where hnRNP A1 differentially binds and regulates RNA, including aberrantly spliced targets identified in human samples. Additionally, dysfunctional hnRNP A1 expression in neurons caused neurite loss and identical changes in splicing, corroborating hnRNP A1 dysfunction as a cause of neurodegeneration. Collectively, these data indicate hnRNP A1 dysfunction causes altered neuronal RNA splicing, resulting in neurodegeneration in MS.

## Abstract (web summary)

HnRNP A1 dysfunction is associated with neurodegeneration in multiple sclerosis (MS). Herein, advanced RNA sequencing and CLIPseq of MS brains and relevant models demonstrated that hnRNP A1 binding of target RNAs and RNA splicing were altered, precipitating neurodegeneration.

## Introduction

Neurodegeneration, the damage to and loss of neurons and axons, drives multiple sclerosis (MS) disease severity and disability^1,2^. Substantial neuronal loss, decreased cortical volume, and axonal transection are observed in both MS gray matter as well as cortical plaques^1–6^. MS neurodegenerative pathology is marked by multiple parallel processes, including inflammation, synaptic loss, glutamate excitotoxicity, and mitochondrial dysfunction^7–14^. However, RNA binding protein (RBP) dysfunction has become a defining feature of neurodegeneration in numerous other diseases, including amyotrophic lateral sclerosis, frontotemporal dementia, Alzheimer’s disease, and Huntington’s disease^15–22^. In these diseases, RBP dysfunction is identified through RBP mislocalization, inherited and somatic mutations within RBPs, RBP aggregation, and changes in RBP expression. These cumulatively lead to widespread changes in RNA metabolism, including disrupted binding of target RNA, and alterations in splicing, nonsense-mediated decay, and RNA transport^23–29^. Aberrant changes in these processes are detrimental to cells, resulting in damage to or loss of neurons and subsequent neurodegeneration. Interestingly, sequencing studies from MS plaques and an animal model of MS, experimental autoimmune encephalomyelitis (EAE), show significant enrichment for processes related to RNA metabolism, such as ribonucleotide metabolism, translation, and RNA binding^30–32^, implicating an important role for RBP dysfunction and downstream processes in MS pathogenesis. Other studies reveal a significant role for changes in splicing, a process highly regulated by RBPs, in MS^32–34^. We recently showed RBP dysfunction is a dominant feature of neurons from MS cortex^35^, neurons from mice with EAE^36,37^, and relevant in vitro models^38–40^. Specifically, we identified the RBP heterogeneous nuclear ribonucleoprotein A1 (hnRNP A1), which binds a multitude of RNA targets, as severely dysfunctional in these systems. These data, combined with evidence of disruption to RBP-related processes in MS, suggest RBP dysfunction may be a novel mechanism contributing to neurodegeneration in MS.

In this study, we characterized the consequences of hnRNP A1 dysfunction on RNA metabolism in MS cortex and EAE using both total RNA sequencing (RNAseq) and UV-crosslinking immunoprecipitation followed by RNA sequencing (CLIPseq). Because human cortex from progressive MS patients exhibits substantial hnRNP A1 mislocalization^35^, we performed RNAseq comparing human brain cortical samples from healthy controls with those from progressive MS patients to identify pathways and alternative RNA splicing events that may be impacted by hnRNP A1 dysfunction. Subsequent CLIPseq experiments employing naïve and EAE mice identified dramatic changes in the RNA binding profile of hnRNP A1 during disease, including differentially expressed and differentially spliced targets that were identified in our human samples. We confirmed sequencing-identified alternative splicing events within multiple RNA targets, verified that these alternative splicing events also occurred in EAE mouse spinal cord, and demonstrated that these alternatively spliced RNA isoforms were destined for nonsense-mediated decay. Further, to directly connect neuronal hnRNP A1 dysfunction to these events, we transduced primary embryonic mouse cortical neurons with dysfunctional mutant hnRNP A1, resulting in neurite loss and confirmation of RNA splicing changes, which was duplicated with CRISPR/Cas9 knockout of hnRNP A1. We also showed that hnRNP A1 bound alternatively spliced RNA within the CLIPseq-identified region by EMSA (electrophoretic mobility shift assay). Thus, herein we have established a solid foundation of work indicating that neuronal hnRNP A1 dysfunction in MS disrupts RNA metabolism, and we provide mechanisms by which this promotes neurodegeneration in MS and relevant models.

## Results

### MS cortex with mislocalized neuronal hnRNP A1 has an altered RNA expression profile indicative of neurodegeneration

**Fig. 1:**
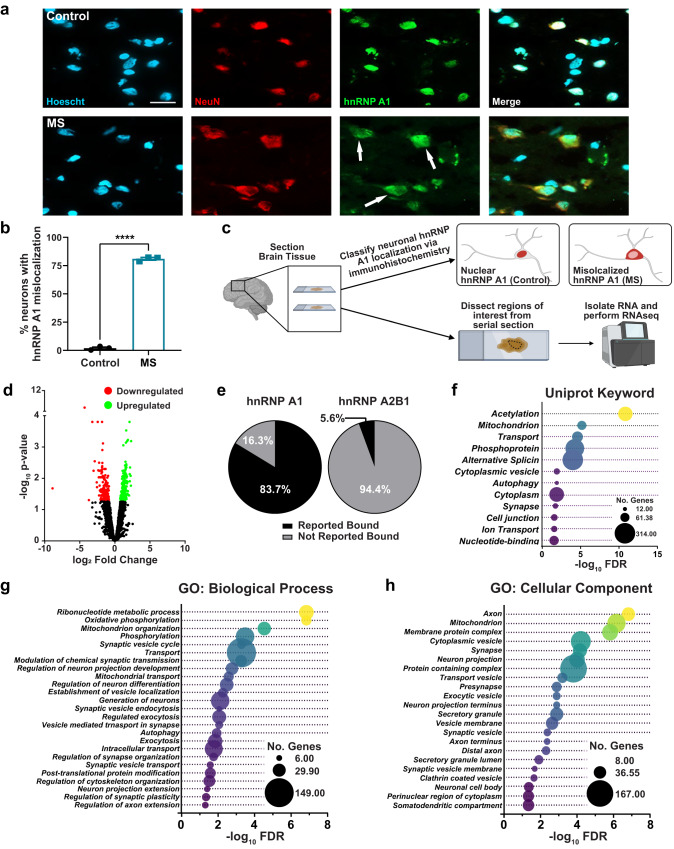
MS cortex exhibiting mislocalized neuronal hnRNP A1, has an altered RNA expression profile indicative of neurodegeneration. **a**, **b** Neurons (NeuN, red) from control cortex demonstrate predominantly nuclear (Hoechst, blue) localization of hnRNP A1 (green), whereas significantly more neurons from MS cortex exhibit hnRNP A1 cytoplasmic mislocalization (arrows). Scale bar 20 μm. Unpaired t-test, one-tailed with ****p < 0.0001 with *n* = 3 individual samples per group. Data are graphed as mean ± SEM. **c** Schematic illustrating the workflow for human immunohistochemical and RNA sequencing experiments. Image created with BioRender.com. **d** Volcano plot showing all genes detected by RNAseq. Highlighted are those that were significantly downregulated (red) or significantly upregulated (green) in MS (*n* = 3) as compared to control (*n* = 3) cortex, determined by DESeq2 analysis with a Benjamini–Hochberg adjusted *p*-value of 0.05. **e** Pie charts depicting the percentage of differentially expressed genes that have been previously shown to be bound (black) or not bound (gray) by hnRNP A1 and hnRNP A2B1. **f**–**h** Bubble plots illustrating Gene Ontology (GO) terms that were significantly enriched due to the differentially expressed genes in (**d**) when examining Uniprot Keyword (**f**), GO: Biological Process (**g**), and GO: Cellular Component (**h**). In all plots, bubble size corresponds to the number of differentially expressed genes (No. genes) included within that term while false-discovery rate as a metric of significance (−log_10_FDR) corresponds to the x-axis. Source data are provided as a Source Data file.

We previously demonstrated that neuronal hnRNP A1 mislocalization is a dominant feature of the cortex in MS brains compared to healthy controls^35^. Therefore, we examined the consequences of hnRNP A1 dysfunction in progressive MS brains by employing RNAseq. First, we defined regions with increased neuronal hnRNP A1 mislocalization in MS brains and regions with normal neuronal hnRNP A1 nuclear localization in the control cortex (Fig. 1a, b). RNA was extracted from MS cortex with hnRNP A1 mislocalization and control cortex with normal hnRNP A1 localization, followed by RNA sequencing (Fig. 1c). As previously published, we found that while overall levels of hnRNP A1 expression did not change (Supplementary Fig. 1), significantly more neurons from MS cortex displayed hnRNP A1 mislocalization as compared to controls (Fig. 1a, b). RNA sequencing (Supplementary Table 1) revealed the separation of samples based on condition (Supplementary Fig. 2a, b) and approximately 550 differentially expressed genes between control and MS samples, including 359 upregulated and 192 downregulated genes (Fig. 1d, Supplementary Data 1). Using CLIPdb, a database of direct interactions between RBPs and their target RNA^41,42^, we found that over 80% of differentially expressed transcripts had been previously shown to bind hnRNP A1 (Fig. 1e). In comparison, less than 6% of the differentially expressed transcripts had been previously shown to bind hnRNP A2B1, a structurally similar RBP in the same family as hnRNP A1^43^ (Fig. 1e). This indicates that the majority of the differentially expressed RNAs are likely to be directly regulated by hnRNP A1 and implicates hnRNP A1 cytoplasmic mislocalization as a mediator of altered regulation of these RNAs.

To explore biological processes related to neuronal hnRNP A1 mislocalization in MS brains, we performed gene ontology (GO) enrichment analyses. Here, we found significant enrichment for terms related to alternative splicing, nucleotide binding, and ribonucleotide metabolic processes (Fig. 1f–h, Supplementary Data 2). While hnRNP A1 itself directly carries out these activities, this also suggests MS pathology is hallmarked by major alterations in the regulation of RNAs or other proteins that modulate these processes (Fig. 1f–h). There was also significant enrichment of regulation of axon extension, regulation of neuron projection development, regulation of synapse organization, and regulation of cytoskeleton organization (Fig. 1g), indicating enrichment for processes are important for the maintenance of neuronal structure. This aligns with previous studies showing that the knockdown of hnRNP A1 in neuronal cell lines leads to decreased neurite length and branching^44^. Furthermore, GO component analyses revealed significant enrichment for neuron-specific components, including neuronal cell body, somatodendritic compartment, axon, and synapse (Fig. 1h). Because the majority of genes driving these GO term enrichments are known to be regulated by hnRNP A1, this suggests that dysfunctional hnRNP A1 is responsible for altered regulation of neuronal biological processes and structures. Together, these data suggest a role for hnRNP A1 dysfunction in neurodegeneration via dysregulation of RNAs that are important for neuron and axon maintenance and survival.

### The RNA binding profile of hnRNP A1 is altered in spinal cords of mice with EAE

**Fig. 2:**
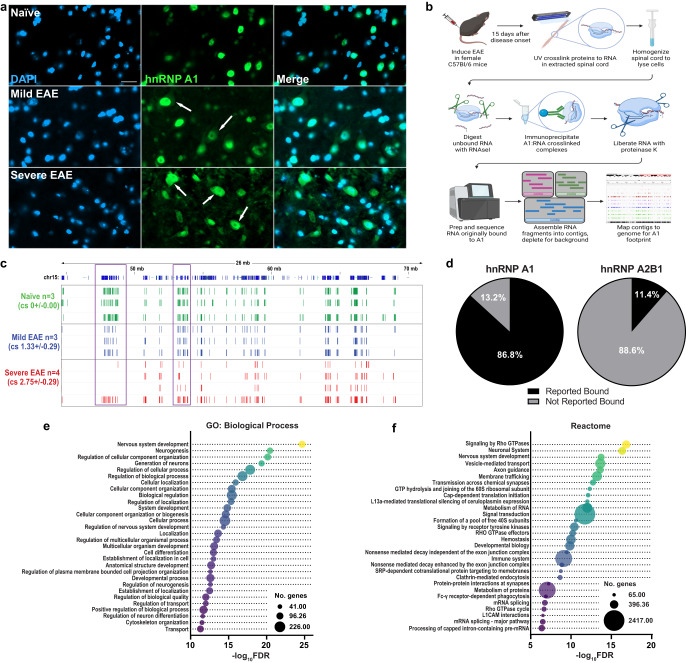
hnRNP A1 CLIPseq of mouse spinal cords detects known and novel hnRNP A1 RNA binding targets. **a** In naïve mice, hnRNP A1 (green) is localized to the nucleus (DAPI, blue) of spinal cord neurons. In contrast, mice with both mild and severe EAE demonstrate hnRNP A1 cytoplasmic mislocalization (arrows) in spinal cord neurons. Scale bar 20 μm. **b** Schematic describing the CLIPseq workflow. Image created with BioRender.com. **c** EAE mice were grouped based on clinical score (cs) where mice with clinical scores greater than 2.5 at the end of the experiment were defined as having ‘severe’ disease, whereas those with clinical scores of 1.0–2.0 were grouped as ‘mild’ disease. The average clinical score of each group (naïve (green) *n* = 3, mild EAE (blue) *n* = 3, severe EAE (red) *n* = 4) is displayed ± standard deviation. An IGV snapshot of chromosome 15 (chr15) illustrates the hnRNP A1 CLIPper footprints for all mice. Purple boxes highlight regions with differential hnRNP A1 CLIPper footprints between groups. **d** The 2312 genes whose RNAs we identified in this study as bound by hnRNP A1 were compared to previously published hnRNP A1 or hnRNP A2B1-bound genes in human datasets (CLIPdb). 305 of the RNAs (13.2%) we identified as bound by hnRNP A1 were not found in CLIPdb, thus classifying them as novel hnRNP A1 RNA targets. Most genes identified as encoding for hnRNP A1-bound RNAs are not bound by hnRNP A2B1. **e**, **f** All hnRNP A1-bound genes we identified were analyzed using GO to establish hnRNP A1-mediated regulation of pathways in mouse spinal cord. Bubble plots illustrate GO terms that were significantly enriched when examining GO: Biological Processes (**e**), and Reactome pathways (**f**). In both plots, bubble size corresponds to the number of bound genes included within that term while false-discovery rate as a metric of significance (−log_10_FDR) corresponds to the x-axis. Source data are provided as a Source Data file.

Our findings in human tissues strongly suggest that the inability of hnRNP A1 to properly regulate and bind RNA in neurons is playing a central role in the pathogenesis of MS. Therefore, we employed CLIPseq in EAE, the most commonly used preclinical in vivo mouse model of MS, to directly assess alterations in the RNA binding profile of mislocalized hnRNP A1. Active induction of EAE in C57Bl/6 mice with myelin oligodendrocyte glycoprotein peptide (MOG_33-55_) leads to the recapitulation of features of MS, including extensive neurodegeneration and neuronal hnRNP A1 mislocalization^36,37,45,46^ (Fig. 2a), which primarily manifest in the spinal cord. We previously showed a significant link between neuronal hnRNP A1 mislocalization and neurodegeneration, including neuronal loss, in EAE at 15 days post-clinical onset^36^, and thus chose to sacrifice mice at this time point. CLIPseq for hnRNP A1 using the spinal cords of mice with EAE (*n* = 7) and age-matched naïve mice (*n* = 3) followed by both traditional peak calling^47,48^ and a de novo assembly approach allowed us to examine how hnRNP A1 bound RNA in naïve and EAE mice (Fig. 2b, Supplementary Fig. 3). We immediately observed that, although sequencing depth and the amount of hnRNP A1-bound RNA was similar among all samples (Supplementary Table 2, Supplementary Fig. 4a), mice with more severe EAE at sacrifice (clinical scores (cs) ≥2.5) demonstrated a distinct hnRNP A1 RNA footprint compared to naïve mice, or mice with mild EAE (cs 1.0–2.0; Fig. 2c). Thus, we assessed hnRNP A1 RNA footprints in three groups based on clinical score (Naïve, n = 3; Mild EAE, *n* = 3; and Severe EAE, *n* = 4).

We used multiple approaches to assess the binding profile of hnRNP A1 in EAE. Initially, we performed an alignment-based approach followed by peak calling using PEAKachu^48^ and CLIPper^47^, the former of which takes biological replicates into consideration while the latter does not. Both methods use size-matched inputs (SMI) as background controls to eliminate or reduce false positives, a well-documented approach^49^. We found that the alignment approaches were potentially biased towards longer reads. Because of this, we also employed an assembly based approach, which assembles contigs from sequencing data^50^. CLIPper identified the highest number of enriched or significant clusters in the greatest number of genes across the animals (Supplementary Fig. 4b, Supplementary Table 3). Despite the differences in the number of genes or significant clusters detected across methodologies, the trend remained the same where there were fewer genes or significant clusters as the disease progressed from naïve to mild EAE to severe EAE (Supplementary Fig. 4b). Of note, one animal within the severe EAE group showed a similar hnRNP A1 RNA binding profile to naïve and mild EAE mice (see Fig. 2c, bottom row). This is unsurprising as EAE is a highly heterogeneous model where identically treated mice demonstrate a wide range of disease severity. This outlier in the severe EAE group had also been in remission two days prior to the end of the experiment, as opposed to the other three replicates, which had developed and maintained a more severe disease course throughout the experiment.

Remarkably, given the variability of the EAE animal model, Irreproducible Discovery Rate (IDR) analysis of CLIPper-identified peaks for samples within each group found largely acceptable reproducibility (Supplementary Fig. 4c)^51^, defined by the ENCODE project as either self-consistency or rescue ratios of less than 2^52^. Of note, the same outlier severe EAE sample with visually different hnRNP A1 binding footprints (Severe EAE 4) was also flagged by IDR as a sample of concern (Supplementary Fig. 4c, table). Given the ENCODE standards were established primarily for clonal cell line-derived samples, the outcomes of this analysis thus support the use of these data for downstream analyses. When comparing genes with an hnRNP A1 footprint across the animals, we found that the majority of PEAKachu-identified genes (>95%) were included within the assembly or CLIPper datasets (Supplementary Fig. 4d). Therefore, we proceeded by analyzing genes common between the assembly and CLIPper methods across the groups to assess the impact of EAE on hnRNP A1-bound genes (Supplementary Data 3).

Although the number of genes and footprint were altered from naïve to mild EAE to severe EAE, hnRNP A1 was still found to bind with specificity to AG-rich regions (Supplementary Fig. 5a)^53,54^. In naïve mice, the previously documented UAG motif^55–57^ was enriched, which corroborates previous findings illustrating the importance of this motif for hnRNP A1 binding. This bolsters the specificity of crosslinking sites and indicates that the identified peaks within genes are hnRNP A1-specific. Interestingly, an AGGU motif is more strongly represented in the EAE samples (Supplementary Fig. 5a), indicating that the binding preferences of hnRNP A1 are altered. Further, we examined hnRNP A1-bound gene regions^58^. Of the reads that aligned to annotated regions, the majority were within intronic regions in all animals (Supplementary Fig. 5b). This echoes what has been previously shown where hnRNP A1 predominantly binds introns^55^, although we observed reduced intronic binding in EAE animals (Supplementary Fig. 5c). Finally, we compared all the RNAs that were identified to be bound by hnRNP A1 in both the CLIPper and assembly datasets to their human counterparts in CLIPdb. We found that a majority (86.8%) of genes were previously identified as hnRNP A1 RNA targets in human non-neuronal cell lines^41^ (Fig. 2d). Importantly, we also identified a total of 305 RNAs (13.2%) that had not been previously reported as hnRNP A1 binding targets (Fig. 2d), and are therefore, novel hnRNP A1-bound RNAs. For comparison, only 11.4% of the RNAs we found to bind hnRNP A1 were reported to be bound by its closest relative, hnRNP A2B1, in CLIPdb (Fig. 2d). When examining all RNAs bound by hnRNP A1 from our CLIPseq data, we found that the top keywords and GO terms (Fig. 2e, f, Supplementary Data 4) are congruent with those identified in MS cortex, highlighting critical RNA metabolic functions like alternative splicing, and nucleotide- and RNA-binding, and major neurological terms related to neuronal and axonal morphology such as axon, dendrite, and synapse.

**Fig. 3:**
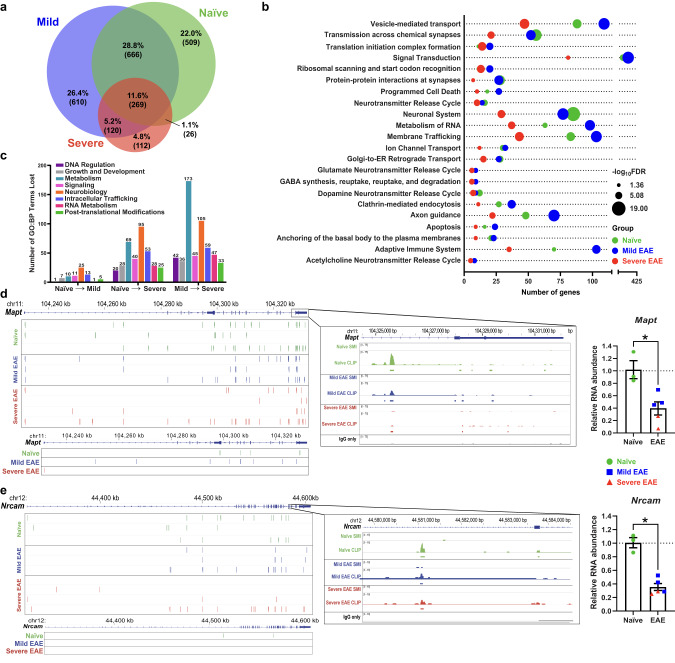
The RNA binding profile of hnRNP A1 is altered in spinal cords of mice with EAE. **a** Volumetric Venn diagram illustrating the percentage of the total hnRNP A1-bound RNAs that are unique and shared between naïve (green, *n* = 3), mild EAE (blue, *n* = 3), and severe EAE (red, *n* = 4) mice. Mice with severe disease bind fewer unique RNAs. **b** To provide a broad functional overview of hnRNP A1-regulated processes in each condition, each set of genes whose RNAs were bound by hnRNP A1 in naïve (green), mild EAE (blue), or severe EAE (red) mice were analyzed for GO enrichment through Reactome Pathways. Terms were sorted by false-discovery rate (bubble size) and plotted against the number of genes contributing to each term (x-axis). Mice with severe disease consistently had fewer genes contributing to each term, with a reduced significance of contribution to regulation of that term. **c** RNAs bound by hnRNP A1 in each condition were assessed separately for GO:BP terms, which were then manually sorted into eight GO:BP categories. Plotted here are the number of terms within each GO:BP category whose regulation were lost (*y*-axis) in transition from naïve to mild through severe EAE. All significant GO:BP terms for each condition were compared, and terms regulated by hnRNP A1 in naïve mice but not in mild EAE mice were defined as lost in naïve → mild. Terms regulated by hnRNP A1 in naïve mice but not in severe EAE mice were defined as lost in naïve → severe. Terms regulated by hnRNP A1 in mild EAE mice but not severe were defined as lost in mild → severe. **d**, **e** To verify the effect of loss of hnRNP A1 binding, two genes (*Mapt, Nrcam*), which were regulated in two or more of the GO:BP categories in (**c**), were selected for further analysis. CLIPper (top) and assembly (bottom) approaches identified hnRNP A1 binding sites within *Mapt* (**d**) and *Nrcam* (**e**) across groups. Inset image is the hnRNP A1 read density in reads per million for naïve, mild EAE, and severe EAE CLIP samples with paired SMI and IgG only controls. CLIPper identified peaks are indicated by boxes below each respective group. qPCR was used to examine changes in expression between naïve and EAE mice. Unpaired Mann–Whitney *U* test, two-way with *p < 0.05 (*Mapt* exact *p* = 0.0357; *Nrcam* exact *p* = 0.0357). Data are plotted as mean ± standard error of the mean for naïve (green circles, *n* = 3) and EAE (mild *n* = 3 blue squares; severe *n* = 2 red triangles) mice. Source data are provided as a Source Data file.

We next compared the total RNAs bound by hnRNP A1 in severe EAE, mild EAE, and naïve samples. Mice from all groups demonstrated both shared and unique hnRNP A1-bound RNAs; however, mice with severe disease bound fewer unique RNAs compared to mild EAE and naive (Fig. 3a). GO analyses using Reactome Pathway analysis^59^ afforded a broad overview of pathways regulated by hnRNP A1 in each group (Fig. 3b). This revealed that while slightly more genes in each term were bound by hnRNP A1 in mild EAE as compared to naïve samples, hnRNP A1 in severe EAE demonstrated a global *loss* of regulation of these terms, concomitant with our earlier observation that severe EAE was marked by hnRNP A1 binding fewer unique RNAs. To assess the consequences of altered hnRNP A1 RNA binding activity as disease progresses, we manually grouped GO:BP terms into categories to identify GO:BP terms that were *lost* between naïve and mild EAE, naïve and severe EAE, and mild and severe EAE (Fig. 3c). Critically, the highest proportion of terms *lost* over disease progression from naïve through mild to severe EAE were involved in neurobiology and cell metabolic functions, closely followed by RNA metabolism, intracellular trafficking, and signaling (Fig. 3c). This indicates that dysfunctional hnRNP A1 no longer has the capacity to properly regulate major cellular processes, a hallmark of RBP-driven neurodegeneration^60–63^.

To verify changes in the binding of hnRNP A1 on relevant RNAs and to assess the impact on target RNA abundance, we selected RNAs that contributed to a high proportion of GO:BP terms *within* and *across* our major categories of interest (neurobiology, intracellular trafficking, growth and development, and RNA metabolism). For example, *Mapt*, a gene heavily associated with neurodegeneration and neuronal tubule stability^64^, demonstrated differential hnRNP A1 binding footprints when comparing naïve and EAE animals, including reduced binding in some regions as per both the CLIPper and assembly approaches (Fig. 3d). We performed RT-qPCR and found a decrease in relative *Mapt* transcript abundance in EAE compared to naïve mice, suggesting that loss of or changes in hnRNP A1 binding and regulation results in decreased transcript stability (Fig. 3d). Similarly, *Nrcam*, a neuronal gene involved in neurite outgrowth and maintenance and synapse formation^65^, showed marked changes in hnRNP A1 binding, especially in severe EAE (Fig. 3e, left) and decreased transcript abundance (Fig. 3e, right). We further confirmed that decreased RNA abundance of these targets was specifically regulated by hnRNP A1 by knocking out hnRNP A1 in a mouse neuronal cell line using transient transfection of CRISPR/Cas9 and short-term selection (Supplementary Fig. 6). We also validated reduced transcript abundance of *Ermn*, *Fam107a*, *Taf1*, *Hnrnpa2b1*, and *Spock3* in EAE mouse spinal cord, as additional hnRNP A1 binding targets highly represented in GO:BP terms (Supplementary Fig. 7). While this assesses abundance of the total RNAs from a given gene and does not explore nuanced isoform variations, this indicates that changes in these transcripts are directly related to hnRNP A1 dysfunction. Thus, altered hnRNP A1 binding results in reduced overall abundance of a given RNA, which may lead to a concomitant loss of protein abundance and function.

Cumulatively, our exploration and discovery of altered hnRNP A1 RNA binding profiles in EAE supports the hypothesis that hnRNP A1 dysfunction, including mislocalization, drive the altered neurodegeneration-associated gene expression we observed in the MS cortex.

### hnRNP A1 dysfunction results in altered neuronal RNA splicing and promotes a neurodegenerative phenotype

A major theme in both datasets was altered RNA metabolic processes, and particularly, alternative splicing. Since alternative splicing regulation is a major function of hnRNP A1^66–70^ and is a key observation in systems that model hnRNP A1 dysfunction^44,71^, we assessed alternative splicing outcomes across both human and mouse systems.

We first examined a known hnRNP A1-regulated alternative splicing event in the gene for pyruvate kinase, *PKM* (human)/*Pkm* (mouse)^72,73^. *PKM* encodes two major isoforms distinguished by mutually exclusive usage of either exon 9 (primary isoform, *PKM1/Pkm1*) or exon 10 (secondary isoform, *PKM2/Pkm2*)^74^. Visualization of RNAseq coverage indicated enrichment of *PKM2* in MS brains (Supplementary Fig. 8a, b). Using LiftOver^75^, a program that converts sequence alignments from one genome (human) to another (mouse) when sequence and exonic architecture are sufficiently conserved, CLIPseq further confirmed differential hnRNP A1 binding of *PKM/Pkm* throughout the gene and within the region of interest (Supplementary Fig. 8a, b) in mouse spinal cord. This was further validated by data from the ENCODE project^69^, which provides CLIP-identified binding sites for numerous RBPs across two biological replicates from two non-neuronal human cell lines (Supplementary Fig. 8a, b). Since exon 10 contains a PstI restriction endonuclease recognition site in both the human and mouse gene, we reverse-transcribed the RNA, PCR-amplified the surrounding exons and digested them with PstI, and visualized the single *PKM1* band *versus* the PstI-cleaved doublet *PKM2* band in human (Supplementary Fig. 8c) and mouse (Supplementary Fig. 8d) samples on an agarose gel. Band densitometry paralleled the sequencing data suggesting that *PKM2* was enriched in MS (Supplementary Fig. 8c), but this did not reach statistical significance due to the variability of human tissues. Considering the visualization of coverage indicating enrichment of *PKM2* in MS brains (Supplementary Fig. 8b), we decided to pursue this in our more controlled mouse model and found that *Pkm2* was clearly enriched in EAE (Supplementary Fig. 8d) samples, confirming changes of an hnRNP A1-regulated splicing event in disease.

To confirm that our observations were both neuron- and hnRNP A1 dysfunction-specific, we established a primary embryonic neuron validation system employing a mutation in the core prion-like domain of hnRNP A1 (F263S), an area which has been associated with hnRNP A1 dysfunction^71,76^. When we transduced primary mouse embryonic neurons with an AAV encoding hnRNP A1(F263S), we found that the mutant dysfunctional hnRNP A1 caused increased accumulation of *Pkm2* (Supplementary Fig. 8e), thus directly confirming that neuronal hnRNP A1 dysfunction leads to the changes in *PKM/Pkm* splicing we observed in MS and EAE. Together, these phenotypes indicate that the F263S mutation induces hnRNP A1 dysfunction resulting in changes in splicing.

**Fig. 4:**
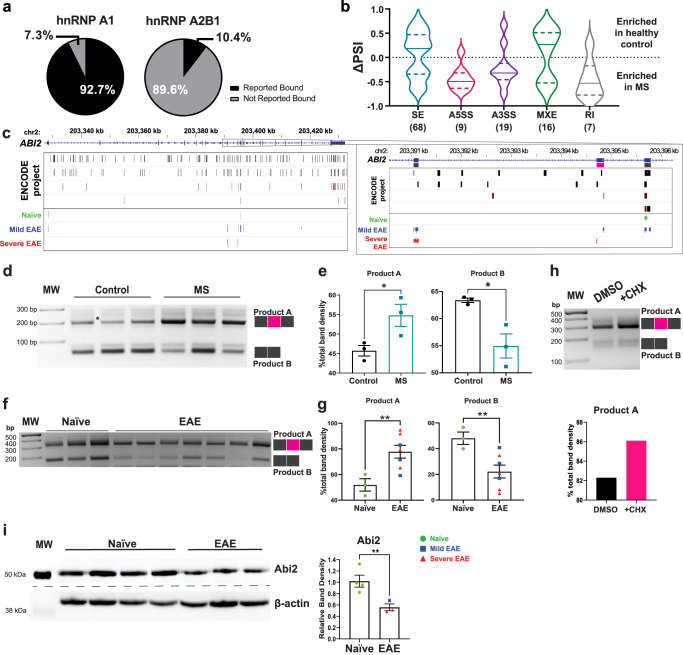
hnRNP A1 dysfunction drives altered neuronal RNA splicing resulting in nonsense mediated decay and decreased protein expression. **a** Percentage of rMATS-identified genes which have been previously shown to be bound by hnRNP A1 or hnRNP A2B1 using CLIPdb, as in Fig. 1e. **b** Violin plot demonstrating the ΔPSI (percent spliced-in) distribution for each alternative splicing event identified by rMATS: skipped exon (SE), alternative 5′ splice site (A5SS), alternative 3′ splice site (A3SS), mutually exclusive exon (MXE), and retained intron (RI), where positive ΔPSI values indicate higher frequency in controls compared to MS. **c** LiftOver was used to overlap unified mouse CLIPseq tracks of hnRNP A1 binding sites on human *ABI2* and combined with data of hnRNP A1 binding sites on human *ABI2* from the ENCODE project to examine the regulation of an rMATS-identified alternative splicing event. **d**, **e** Products were analyzed by PCR using primers to identify the alternative splicing event in the region inset in (**c**) from human samples and detected by gel electrophoresis (**d**). Quantification of PCR products confirmed rMATS findings in which there was a significant increase in the inclusion of the alternative *ABI2* exon (pink from (**c**)) to yield more of Product A in MS cases (*n* = 3) as compared to controls (*n* = 3). Unpaired *t* test, one-tailed with *p < 0.05 (exact *p* = 0.0221). Data are plotted as the mean ± SEM. **f**, **g** Products were analyzed by PCR using primers to identify the alternative splicing event in (**c**) in mouse samples and detected by gel electrophoresis. Quantification of PCR results indicates that EAE mice show a similar change in *Abi2* alternative splicing as was found in MS samples. Unpaired *t* test, one tailed with **p < 0.01 (exact *p* = 0.0077). Data are plotted as the mean ± SEM with naïve (green circles *n* = 3) and EAE (mild, *n* = 3 blue squares; severe, *n* = 4 red triangles). **h** SK-N-SH cells, a human neuronal cell line, were treated with cycloheximide (CHX) (*n* = 1) to inhibit nonsense mediated decay (NMD) or DMSO alone (*n* = 1) followed by PCR analysis of the *ABI2* alternative splicing event. Product A accumulated in the CHX-treated cells indicating it is a product destined for NMD. **i** Given that the inclusion of the alternatively spliced *ABI2/Abi2* exon leads to NMD, we performed western blot for Abi2 in mice to determine if there was concomitant protein loss. EAE mice, which showed increased inclusion of the alternative exon resulting in a NMD destined product, also demonstrated decreased protein levels. Unpaired *t* test, one-tailed with **p < 0.01 (exact *p* = 0.0096). Data are plotted as the mean ± SEM with naïve (green circles *n* = 4) and EAE (mild *n* = 1 blue squares; severe *n* = 2 red triangles). Source data are provided as a Source Data file.

We next employed rMATS, a program designed to identify splicing from RNA sequencing data^77^, to more broadly profile and detect new hnRNP A1-regulated alternative splicing events. This identified 119 significant alternative splicing events in 96 different genes from MS cortex samples as compared to healthy controls. The majority of these genes (>92%) have been previously shown to be alternatively spliced by or bound by hnRNP A1 (Fig. 4a). The highest proportion of alternative splicing events were skipped exons (SE; Fig. 4b), which coincides with proper hnRNP A1 functioning as hnRNP A1 plays a critical role in regulating skipped exons (SEs)^57^. Interestingly, alternative 5′ and 3′ splice site (A5SS and A3SS, respectively) usage and retained introns (RI) were more prevalent in MS compared to controls (Fig. 4b), suggesting that loss of hnRNP A1 function in MS deregulates these events.

We selected multiple splicing events for confirmation using amplicon PCR in relevant hnRNP A1-bound targets, starting with *ABI2*, which encodes a protein (Abi2, Abl interactor 2) involved in cytoskeletal dynamics and dendrite formation. Using LiftOver, we examined our CLIPseq dataset alongside the ENCODE project hnRNP A1-binding footprint and determined that hnRNP A1 binds *ABI2*, including within the region of the alternatively spliced exon (Fig. 4c). We then designed a differential PCR amplification assay to assess exon inclusion: after reverse-transcription, samples were amplified with three PCR primers—a forward and reverse primers in the conserved (black) exons, and an additional reverse primer in the alternative (pink) exon. Resultant products were visualized by gel electrophoresis (Fig. 4d) and assessed for relative inclusion of the middle exon within each lane by band densitometry (Fig. 4e). We thus confirmed the increased inclusion of the alternatively spliced exon in MS as compared to control (Fig. 4d, e). Using the same PCR strategy, we showed that this exon was also retained in EAE *versus* naïve mice (Fig. 4f, g). Since alternatively spliced RNAs are often shunted through nonsense-mediated decay (NMD) for disposal^78^, we next assessed the functional consequence of this alternative splicing in *ABI2*. To do this, SK-N-SH cells, a human neuronal cell line, were treated with cycloheximide (CHX), which inhibits NMD^79,80^, or DMSO as a negative control. Cells treated with CHX showed accumulation of the alternatively spliced *ABI2* product that was found to be increased in MS and EAE samples (Fig. 4h), confirming that the alternatively spliced product was a target for NMD. We carried out western blotting for Abi2 protein to assess the consequences of *ABI2* mRNA NMD, and found concomitantly decreased protein levels in EAE compared to control tissues (Fig. 4i). This indicates that the increased presence of the alternatively spliced *ABI2* product due to hnRNP A1 dysfunction in MS and EAE leads to NMD and resultant decreased protein levels, implicating alternative splicing changes as part of the disease mechanism.

**Fig. 5:**
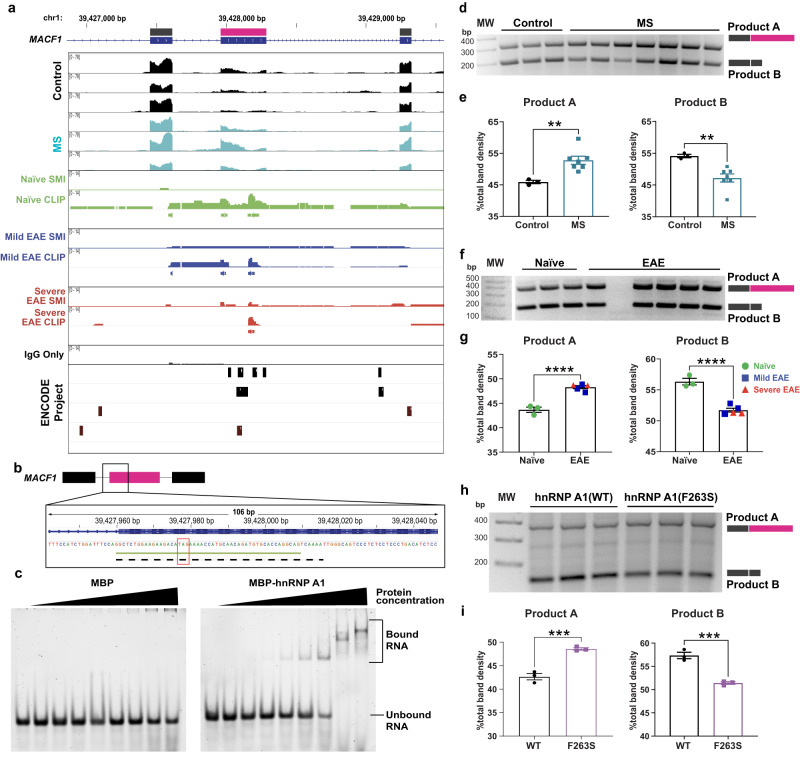
*MACF1/Macf1* splicing is altered in MS and multiple systems of hnRNP A1 dysfunction. **a** RNA sequencing reads in a region of *MACF1* identified by rMATS showing increased reads within the alternatively used exon (pink) in MS as compared to controls. LiftOver was then used to overlap mouse CLIPseq tracks of hnRNP A1 binding sites onto human *MACF1*. LiftOver shows differential hnRNP A1 binding within the area of interest in naïve *vs*. EAE samples, with hnRNP A1 read density in reads per million for naïve, mild EAE, and severe EAE CLIP samples, each shown with paired SMI and IgG only controls. CLIPper-identified peaks are indicated by boxes below each respective animal. Data of hnRNP A1 binding sites in human *MACF1* from the ENCODE project were incorporated to confirm binding sites from previously published datasets. **b** The alternatively spliced exon of *MACF1* contains a UAG motif (red box, known to be preferentially bound by hnRNP A1. The light green line is indicative of the naïve hnRNP A1 binding footprint. The dotted black line is the *MACF1* RNA used for (**c**). **c** RNA-EMSA comparing the binding capacity for *MACF1* (from (b)) between recombinant MBP-tagged hnRNP A1 and MBP alone, confirming that the tag does not contribute to binding. A representative experiment of *n* = 3 independent experiments is shown. **d**, **e** Products were analyzed by PCR using primers to identify the alternative splicing event in (a) from human samples and detected by gel electrophoresis (**d**). **e** Quantification of PCR products confirmed rMATS findings in which there was a significant increase in the inclusion of the middle *MACF1* exon (pink from (**a**)) to yield more of Product A in MS cases (*n* = 7) as compared to controls (*n* = 3). Unpaired *t* test, one-tailed with **p < 0.01 (exact *p* = 0.0050). Data are plotted as the mean ± SEM. **f** Products were analyzed by PCR using primers to identify the alternative splicing event in (**a**) in mouse samples and detected by gel electrophoresis. **g** Quantification of PCR results indicates that EAE mice show a similar change in *Macf1* alternative splicing as was found in MS samples. Unpaired *t* test, one-tailed with ****p < 0.0001. Data are plotted as the mean ± SEM with naïve (green circles *n* = 3) and EAE (mild, *n* = 3 blue squares; severe, *n* = 2 red triangles) mice. **h**, **i** Products were analyzed by PCR to identify the same *Macf1* alternative splicing in primary neurons transduced with wild-type (WT) (*n* = 3 biological replicates) or dysfunctional hnRNP A1(F263S) (*n* = 3 biological replicates) and detected by gel electrophoresis (**h**). **i** Neurons transduced with dysfunctional hnRNP A1 show the same significant changes in exon usage within *Macf1* as EAE and MS samples. Unpaired *t* test, one-tailed with ***p < 0.001 (exact *p* = 0.0007). Data are plotted as the mean ± SEM. Source data are provided as a Source Data file.

Of the alternative splicing events identified by rMATS, we noted that three occurred within microtubule-actin crosslinking factor 1 (*MACF1*) (Supplementary Fig. 9a). MACF1 is a cytoskeletal protein within the CNS that is critical for intraneuronal trafficking, implicated in proper synaptic functioning^81,82^, and has been shown to be significantly changed in vulnerable neuronal populations in MS patients^30^. Furthermore, changes in splicing that result in different isoforms of *MACF1* have been associated with neurodegenerative disease^83^ and disorganized brain structure^84^. Our CLIPseq analyses in mouse spinal cord and data from the ENCODE project confirmed that hnRNP A1 binds *MACF1*/*Macf1* (Supplementary Fig. 9a). Using LiftOver, hnRNP A1 binding sites were identified within regions of the alternative splicing events (Supplementary Fig. 9a). Therefore, we explored one of the alternative splicing events in our human and mouse systems, which had a substantial hnRNP A1 footprint (Fig. 5a). In this region, sequencing data showed increased reads within the exon of interest in MS samples as compared to controls, indicating it was more prevalent in the MS samples (Supplementary Fig. 9b). Further, the alternatively spliced exon contains a UAG motif (Fig. 5b), which is a known hnRNP A1 binding motif that we confirmed in our CLIPseq datasets (Supplementary Fig. 5).

To confirm that hnRNP A1 specifically binds the target MACF1 RNA sequence, we performed RNA electrophoretic mobility shift (RNA-EMSA) with *MACF1*-derived RNA (Fig. 5b, black dotted line) and recombinant hnRNP A1, which was tagged with maltose binding protein (MBP) to prevent the protein from aggregating. Here, we found that recombinant hnRNP A1 bound *MACF1* RNA, but MBP alone did not (Fig. 5c). Next, we used differential PCR as described above for *ABI2* to verify the *MACF1*/*Macf1* splicing event (Fig. 5d, e). We found increased levels of the product containing the exon of interest (Product A) and decreased levels of the product without the exon of interest (Product B) in MS samples as compared to controls. We further observed differential binding in the alternatively spliced exon in EAE mice compared to naïve (Fig. 5a) in our CLIPseq dataset, so we used the same PCR strategy to determine that retention of this same exon was also enriched in *Macf1* RNA from EAE mouse spinal cords (Fig. 5f, g).

Finally, we confirmed that this alternative splicing event was both neuron- and hnRNP A1-specific by carrying out the same PCR-based assay in two additional systems of hnRNP A1 dysfunction: primary mouse embryonic neurons transduced with hnRNP A1(WT) or mutant dysfunctional hnRNP A1(F263S) both tagged with the mCherry fluorescent reporter (Fig. 5h, i) and hnRNP A1 knockout cells described above (Supplementary Fig. 9c, d). The abundance of the retained exon was once again augmented in cells transduced with dysfunctional hnRNP A1(F263S) and those with hnRNP A1 knockout. MACF1 controls actin organization and dynamics and thus, may be critical for neuronal process development and maintenance. Therefore, we examined neurites, which could be dysregulated due to changes in *Macf1* splicing, in hnRNP A1(F263S)-transduced neurons compared to wildtype-transduced neurons (Supplementary Fig. 9e). Here, we found a significant reduction in neurite length in neurons transduced with mutant hnRNP A1 as compared to wildtype.

Together, our findings in MS brains, EAE spinal cords, primary neurons expressing dysfunctional hnRNP A1, and hnRNP A1 CRISPR knockout mouse neuron-like cells, lead us to conclude that hnRNP A1 dysfunction promotes neurodegeneration by altering binding and splicing of RNAs critical for neuron function.

## Discussion

RBP dysfunction, including mislocalization, mutations within RBPs, aggregation, and changes in expression are pathologic hallmarks of neurologic diseases^22,76,85–89^. While RBP dysfunction may manifest through one or several of these pathways in disease, there is a common convergence resulting in changes in RNA metabolism^90^. This is crucial in neurons, which have lower rates of RNA turnover due to their longevity, an increased need for highly regulated local translation in axons and dendrites, and interconnectivity^91^. Our understanding of the impact of dysfunctional RBPs on neurodegeneration in neurologic disease has exponentially expanded in the last decade. However, the role of RBP dysfunction in MS, a neurologic disease with a significant neurodegenerative component, has remained largely unexplored. Our previous studies indicate that neuronal dysfunction of hnRNP A1, an RBP that plays major roles in RNA metabolism, is characteristic of MS cortex and related models, including EAE (the most commonly used preclinical model of MS), thus implicating hnRNP A1 dysfunction as a component of neurodegeneration in MS^15,35–40,43,44,46,92–94^.

Here, we characterized the significant role hnRNP A1 dysfunction plays in MS and EAE-associated neurodegeneration through its disrupted binding and changes in the splicing of RNA targets involved in neuronal processes. First, using RNA sequencing of MS tissues with neuronal hnRNP A1 mislocalization, we found almost 600 differentially expressed transcripts, the majority of which have been previously found to bind hnRNP A1 in humans. Several processes related to alternative splicing, RNA metabolism, neuronal maintenance, and axonal health were enriched in the dataset. Other sequencing datasets from MS and related models have previously shown significant enrichment for processes tied to RNA metabolism, including ribonucleotide metabolism, translation, nuclear mRNA splicing, and RNA binding^31–33^. This supports our conclusions that changes in RNA metabolism are a hallmark of MS pathogenesis, and we further note that hnRNP A1 dysfunction has been shown to affect these pathways across numerous model systems^44,71,86,95^.

Next, we used CLIPseq of EAE spinal cords to directly link hnRNP A1 dysfunction to the changes observed in MS samples. CLIPseq identified more than 300 novel hnRNP A1-specific targets in addition to paralleling previously documented hnRNP A1-RNA interactions from human CLIP studies^41^. Naïve mice showed a substantial hnRNP A1-specific RNA binding profile, which was altered concomitant with EAE disease severity, suggesting dysfunctional hnRNP A1 RNA binding and regulation as the disease progresses. These data align with our previous studies showing a relationship between neuronal hnRNP A1 dysfunction, hallmarked by its mislocalization, and EAE severity^36^. Studies in other neurodegenerative diseases mimic these results where increased RBP mislocalization, an indicator of dysfunction, correlates with more severe disease phenotypes and progression^96–98^. Importantly, we found that as the disease progressed, hnRNP A1 lost the ability to properly bind RNAs related to vital neuronal and RNA metabolic processes, which were significantly enriched in our human dataset. Furthermore, differential hnRNP A1 binding negatively impacted the abundance of targeted RNAs, suggesting that hnRNP A1 dysfunction may directly impact neurodegeneration via loss of proper target RNA regulation.

Considering altered RNA metabolism was a major finding in both our human and mouse datasets, and given the plethora of studies illustrating the broad impact hnRNP A1 has on splicing^57,67,68,70,99^, we focused on hnRNP A1 and RNA splicing as an indicator of its dysfunction, and as a potential mediator of neurodegeneration. Using rMATS, we found that changes in splicing were skewed towards exon-skipping events, which is supported by findings with other dysfunctional RBPs that lead to increased exon inclusion and subsequent NMD of target transcripts^26,27,29,100–102^. Homeostatic hnRNP A1 typically represses alternative exon inclusion due to its preference for binding and blocking splice enhancer sequences in introns and alternative exons^43^, and CLIP studies in cell lines found increased hnRNP A1 binding in areas near alternatively spliced exons^57^. Altogether these data support a role for hnRNP A1 RNA binding changes and dysfunction in driving increased exon inclusion phenotypes.

With this in mind, we assessed the effect of hnRNP A1 dysfunction on alternative splicing in three different targets identified in our human RNAseq dataset, which were also bound by hnRNP A1 in our CLIPseq dataset: one was a previously known alternative exon substitution (*PKM*)^72–74,103–107^, while two were *novel* exon inclusion events (*ABI2, MACF1*) identified by rMATS. We confirmed each of these alternative splicing events from our human brain samples by PCR, verified that they also occurred in EAE spinal cord, and then confirmed that they were directly caused by hnRNP A1 dysfunction using primary neurons transduced with dysfunctional hnRNP A1, or hnRNP A1 knockout in neuronal cells. We additionally demonstrated that hnRNP A1 directly binds the region of interest in *MACF1*. Finally, we established a phenotypic outcome of this dysregulation: exon inclusion resulted in nonsense-mediated decay of the target mRNA (*ABI2*), and concomitant loss of protein abundance.

Alternative splicing of each of the targets we investigated has known implications for neuronal and brain homeostasis. Increased energy metabolism and overall *PKM* abundance in MS cortical lesions have previously been found via single-nucleus RNA sequencing^30^, while the alternative isoform has also recently been found to translocate to the nucleus and directly impact histone phosphorylation and transcription factor activity to modulate RNA transcription^74^. Another *Abi2* exon inclusion RNA isoform was activated during neuronal differentiation from a mouse teratoma cell line and subsequently found to be enriched in mouse brain^108^; Abi2 itself has been shown to be critical in neuronal cytoskeletal dynamics^109^. *MACF1* is highly expressed in the nervous system and is responsible for cytoskeletal stability through crosslinking of actin and microtubules. Critically, changes in isoform usage for *MACF1* have been found to impact both synaptic and neuronal structural integrity^81–84,110^. Other changes in the splicing of neuronal targets may have similar effects in disease. A recent EAE study found increased exon inclusion events in neurexins 1-3 (*Nrxn1-3*), a set of synaptic proteins^34^, which we identified as differentially bound by hnRNP A1 in EAE.

Here we assessed altered RNA abundance and splicing in the brains of people with MS, and in the spinal cords of mice with EAE. Several other studies examined MS-associated alternative splicing using peripheral blood mononuclear cells, blood samples, serum, and cerebrospinal fluid, while far fewer have directly examined brain tissues from MS patients^111–114^. Further, many of the previously identified targets are related to the immune response, which is unsurprising given the autoimmune component of disease^111^. To our knowledge, this is the first study using RNA sequencing data from MS cortex to examine neuron-specific alternative splicing events, and to use these events to bridge dysfunction of a specific RBP with MS-specific neurodegeneration. We linked dysfunction of the RNA binding protein hnRNP A1 with pathology in both MS and EAE and demonstrated a causal relationship between hnRNP A1 dysfunction in primary neurons and neurodegenerative phenotypes. Finally, we used our experimental systems to identify specific RNA alterations that mechanistically connect hnRNP A1 dysfunction to neurodegeneration. Between differentially expressed RNAs, alternatively spliced transcripts, and altered interactions between hnRNP A1 and numerous RNAs in MS and EAE, we identified thousands of changes in RNAs that may contribute to hnRNP A1 dysfunction-mediated neurodegeneration. Given this, we hypothesize that MS- and hnRNP A1 dysfunction-associated neurodegeneration is not driven by alteration of a single mRNA transcript, but instead is a product of multiple mRNA alterations accumulating over months and years of dysfunction that are likely to have severe consequences for neuronal viability. Altogether, this work represents the first definitive demonstration of RBP dysfunction contributing to neurodegeneration in MS.

## Methods

The research performed in this manuscript complies with all relevant ethical regulations. All human autopsy and animal experiments were approved under either the University of Saskatchewan Biomedical Research Ethics Board (BIO #17-207) or the University of Saskatchewan Animal Research Ethics Board under Animal Use Protocol AUP20170104.

### Cases and autopsy material

Fresh frozen autopsy material from 5 MS cases and 5 control cases with no known neurological deficits was used (Supplementary Table 4). Clinical details were available for many, but not all, cases.

### Immunohistochemistry

Fresh frozen human samples were cryosectioned at 10 μm before being fixed in ice cold acetone at −20 °C for 10 min. Following fixation, sections were allowed to air dry before being washed in PBS. Endogenous peroxidase activity was blocked using 0.3% hydrogen peroxide diluted in methanol. Sections were then blocked in 10% FBS diluted in PBS for 1 h at room temperature, before being placed into primary antibody overnight at 4 °C. Following washes, sections were incubated in the appropriate biotinylated IgG secondary antibodies for 1 h at room temperature. The Vector ImmPRESS DAB kit (Vector Laboratories MP-7802-15) was then used to develop sections. After development, sections were counterstained with hematoxylin and mounted. For immunofluorescence, sections were fixed as above followed by treatment with TrueBlack Lipofuscin Autofluorescence Quencher (Biotium 23007). Sections were blocked and incubated with primary antibody overnight at 4 °C. After PBS washing the next day, slides were incubated with fluorescent-conjugated secondary antibodies for 1 h at room temperature. During final washes, Hoechst was added to stain nuclei. Sections were coverslipped using ProLong Gold antifade mountant. The following antibodies were used: mouse anti-hnRNP A1 (clone 4B10; Millipore; 1:1000), rabbit anti-NeuN (Abcam ab177487; 1:1000), donkey anti-mouse Alexa Fluor 488 (Jackson Immunoresearch 715-546-151; 1:1000, donkey anti-rabbit Alexa Fluor 594 (Jackson Immunoresearch 711-586-152; 1:1000).

### Quantitative pathological assessment of hnRNP A1 mislocalization

3,3′-Diaminobenzidine (DAB)-stained sections were quantitatively assessed for neuronal hnRNP A1 mislocalization based on previously published criteria defining hnRNP A1 localization patterns in MS tissues^35^. Briefly, neurons with robust nuclear signal were classified as normal nuclear localization, while neurons with decreased nuclear signal and hnRNP A1 nucleocytoplasmic mislocalization were classified as positive for hnRNP A1 mislocalization. At least 200 neurons from 15 different fields of view were used for quantification. Images for quantification were acquired using an Olympus UPlanFLN 40X/0.75 numerical aperture objective on an Olympus BX53 scope.

### Human RNA sequencing

#### RNA extraction from human tissues

Areas with hnRNP A1 nuclear localization (controls) and hnRNP A1 mislocalization (MS) were identified using immunohistochemistry. 10 μm cryostat serial sections were used to dissect out regions of interest, which were placed into extraction buffer from the Arcturus PicoPure extraction kit and processed for RNA extraction. Briefly, tubes were vortexed to submerge tissue in extraction buffer before being placed into a shaking incubator at 500 rpm set to 42 °C for 30 min. Samples were centrifuged for 2 min at 3000 × *g* and the supernatant was collected into a new tube. 70% ethanol was added and mixed until a homogenous mixture was achieved. Conditioning buffer was used to prime RNA purification columns and RNA was loaded onto the column followed by centrifugation and several washes. RNA was eluted and stored at −80 °C.

#### RNA library preparation and sequencing

Similar to previous publications^44,46^, 10 ng of input RNA was used for library preparation using the Ovation SoLo RNA-Seq Library Preparation Kit (Tecan Genomics 0500-32) per the manufacturer’s instructions, which included an rRNA depletion step using the AnyDeplete Human Probe Mix. Libraries were quantified using a Qubit 4.0 Fluorometer and Qubit 1X dsDNA HS assay before being run on a TapeStation 4150 to assess library quality. Barcoded libraries were pooled equimolar and run on a NextSeq550, generating 75-bp paired-end reads. Reads were extracted and adapter-trimmed using bcl2fastq (version 2.19.0.316; Illumina), and additional quality trimming was performed using fastp^115^ with default settings except for “-f 5 -Y 0 -g”. Trimmed reads were aligned to the GRCh38.p12 human reference genome (GENCODE Release 30) using STAR^116^ (version 2.5.1b) with default settings. Aligned reads were deduplicated using NuDup (version 2.3.3; Tecan Genomics) with default settings and gene-level expression quantified using htseq-count from the HTSeq package^117^ (version 0.11.3). Genes that were differentially expressed between control and MS samples were identified using DESeq2Shiny Software^118^. Due to disparities in sex between control and MS samples (Supplementary Table 4), known human sex-associated genes^119^, which could skew results, were removed from differential expression analyses. Genes were considered differentially expressed at a Benjamini-Hochberg adjusted p-value threshold of 0.05.

#### Gene ontology and transcript binding analysis

Gene ontology (GO) analyses were performed with the resultant differentially expressed genes using Cytoscape Software (v. 3.8.2)^120^, including the STRING database and STRING enrichment analysis software^121^. CLIPdb contains information on binding sites from CLIPseq experiments on over 300 RBPs^41,42^, so the CLIPdb module was used to search for known RNA binding partners of hnRNP A1 and hnRNP A2B1 in human cells, and the results were exported and used for comparison with the identified differentially expressed transcripts.

#### Identification of alternative splicing targets from RNA sequencing data

Changes in splicing were examined using rMATS (version 4.1.2), which identifies splicing events from RNA sequencing datasets^77^. The aligned, deduplicated RNAseq reads noted above were used with default settings with the read length set to 71 and using paired read analysis.

### Experimental autoimmune encephalomyelitis

All animal experiments were performed in accordance with the University of Saskatchewan’s Animal Research Ethics Board under Animal Use Protocol AUP20170104. Mice were housed five to a cage under pathogen-free conditions on a 12 h/12 h light:dark cycle with *ad libitum* access to standard rodent chow and water. Adult female C57BL/6 N mice (10 weeks) were purchased from Charles River Laboratories. EAE was induced as previously published through active immunization with reagents purchased from Hooke Laboratories (cat. no. EK-2110)^36,37,46,93^. At 12 weeks of age, mice were subcutaneously immunized with 0.1 mL emulsion of MOG_35-55_ in complete Freund’s adjuvant between both the shoulder blades and hind limbs. On days 0 and 1, mice also received 100 ng of pertussis toxin diluted in PBS intraperitoneally. Following induction, mice were evaluated daily for the appearance of clinical signs. Mice were assigned clinical scores based on disease severity according to the scoring scale established by Hooke Laboratories. We have previously established that EAE mice display significant neuronal hnRNP A1 dysfunction at 15 days post-clinical onset in the spinal cord, which is the area of the CNS affected in this model^36^. Therefore, mice were sacrificed at 15 days post-clinical onset with an overdose of Euthanyl for further analyses.

### Immunofluorescence

Animals sacrificed for immunofluorescent analysis were perfuse fixed with 4% paraformaldehyde. Following a 48-h post-fixation period, tissues were put through a sucrose gradient and embedded in OCT. Sections were cut at 10 μm and stored at −80 °C until further use. For staining, sections were thawed at room temperature followed by PBS washing. Sections were blocked using 100% SeaBlock Blocking Solution (ThermoFisher) for 1 h at room temperature before being placed into primary antibody and incubated overnight at 4 °C. Secondary antibodies were incubated with sections for 1 h at room temperature the following day and then mounted using ProLong Gold. The following antibodies were used: mouse anti-hnRNP A1 (clone 4B10; Millipore) and donkey anti-mouse Alexa Fluor 488 (Jackson Immunoresearch).

### CLIPseq

#### Spinal cord extraction and UV crosslinking

Following euthanasia, the spinal column was immediately removed and the spinal cord was hydraulically extruded through the cervical opening with sterile, RNase-free PBS as described by Richner et al.^122^. The cord was then diced into <1mm^3^ fragments in a petri dish with 5 mL ice cold RNase-free PBS and crosslinked in a Spectronics Spectrolink UV Crosslinker with two doses of 400mJ 254 nm UV light, swirling in between doses. Fragments were then collected into a 15 mL conical tube in RNase-free PBS.

#### Spinal cord lysis and immunoprecipitation

The following was carried out with RNase-free reagents, under RNase-free conditions, on ice unless otherwise noted, and is based upon the eCLIP RNAseq method and buffers described by van Nostrand et al.^49^. In brief, spinal cord fragments were pelleted and re-suspended in iCLIP lysis buffer^49^ containing RNase and protease inhibitors, then homogenized using a Tissue Tearor homogenizer and incubated on ice for 30 min to complete lysis. After pelleting tissue debris, supernatant was collected and assessed for protein concentration, and 2 mg protein per sample was prepared for immunoprecipitation with 50 µL Pierce magnetic protein A/G beads (Fisher Scientific Canada PI88803) and 2 µg mouse anti-hnRNP A1 (clone 4B10, Millipore Canada). 2 mg pooled naïve and pooled EAE samples were also prepared for immunoprecipitation with 2 µg of a control mouse IgG2b antibody (clone GC198, Millipore Canada). Samples were treated with Turbo DNAse (Ambion; Fisher Scientific Canada) and RNase I (Fisher Scientific Canada) at a concentration of 1:250 (Supplementary Fig. 10) for 5 min at 37 °C, then incubated with beads and antibody overnight with rotation at 4 °C.

#### End-healing and extraction of RNA from immunoprecipitations

The following was carried out with RNase-free reagents, under RNAse-free conditions, on ice unless otherwise noted, and is based upon the eCLIP RNAseq method and buffers described by van Nostrand et al.^49^. In brief, after overnight immunoprecipitation, two aliquots of 2% volume were collected from each sample as RNA and protein input samples. The remainder of the samples were magnetized and washed with a low-salt wash buffer^49^, then end-healed with polynucleotide kinase (PNK; New England Biolabs Canada) in two steps: first, at pH 6.5 in the absence of ATP to repair the 2′,3′-cyclic phosphate end of the RNA left by Rnase I, and then at a pH of 7.6 supplemented with 1 mM ATP (New England Biolabs Canada) to repair the 5′-hydroxyl end of the RNA left by Rnase I. Samples were magnetized and washed with low- and high-salt wash buffers^49^, and separated into two aliquots: 20% volume was magnetized and collected into 2X SDS sample lysis buffer for western blot analysis, while 80% volume was treated with proteinase K to liberate the RNA from crosslinked protein for sequencing. Samples were then purified with an equal volume of phenol:chloroform:isoamyl alcohol (25:24:1; Millipore Canada) and centrifuged using QuantBio phase-lock gel heavy tubes (VWR Canada), and the aqueous phase was subjected to RNA cleanup using the Zymo RNA Clean & Concentrate-5 kit according to the manufacturer’s protocol. RNA input samples were also subjected to phenol:chloroform:isoamyl alcohol purification and RNA cleanup as above, and then were treated with PNK as described above, and purified using MyOne Silane beads (Dynabeads, Thermo Scientific Canada) as described in van Nostrand et al.^49^. All samples were eluted into 12 μL nuclease-free water for CLIPseq RNA library preparation.

#### CLIPseq RNA Library Preparation

Input and immunoprecipitation samples were prepared for sequencing with Rnase-free reagents, under Rnase-free conditions, and on ice, using the NEXTFlex Small RNA Sequencing Kit v3 (Perkin Elmer; D-mark Biosciences) according to the manufacturer’s protocol, with the recommended “No Size Selection Protocol” modification for bead cleanup, and using PAGE Size-selection and Cleanup to extract library DNA between 150-170nt in length. Sequencing libraries were quantified using a Qubit 4.0 fluorometer (Invitrogen, Thermo Fisher Scientific, Waltham, MA, USA) and Qubit 1x dsDNA HS assay (Invitrogen). The library fragment length distributions were determined using a TapeStation 4150 instrument (Agilent, Santa Clara, CA, USA) with High Sensitivity D1000 ScreenTape and reagents (Agilent). The barcoded libraries were pooled equimolar and 76 bp or 38 bp paired-end reads generated on a NextSeq 550 instrument (Illumina, San Diego, CA, USA).

#### irCLIP

Prior to irCLIP experiments, the oligonucleotide adaptor^123^ was conjugated to a fluorophore for visualization of hnRNP A1-bound RNA as described previously^123^. Briefly, the lyophilized oligonucleotide was acquired from IDT (5′-Phos-CAAGCAGAAGACGGCATACGaaaaaaaaaaaa/iAzideN/AAAAAAAAAAAA) and reconstituted in 500 μL nuclease-free water. The adaptor was adenylated using the NEB 5’ DNA adenylation kit (New England Biolabs Canada) for 2 h at 65 °C followed by inactivation for 10 min at 85 °C. The resultant reaction was precipitated overnight at −20 °C with 3 M sodium acetate and 100% ethanol. The precipitated adenylated oligonucleotide was pelleted at 16,000 × *g* for 30 min at 4 °C. The pellet was washed with 80% ice-cold ethanol and allowed to air dry before being resuspended in 180 μL PBS. Using click chemistry, the adaptor was conjugated to IRdye-800CW-DBCO (LI-COR Biosciences) and column-purified using the QIAquick nucleotide removal kit (Qiagen). irCLIP was performed as previously described with modifications^123,124^. Crosslinked spinal cord lysates were treated with RNase, immunoprecipitated, and treated with PNK as described above. Following PNK treatment, samples were washed and incubated with the fluorescent adaptor overnight at 16 °C shaking at 1100 rpm. Samples were washed and RNA:protein complexes were dissociated in 1X NuPAGE protein loading buffer by incubating at 80 °C for 5 min. Samples were separated on a 4-12% NuPAGE Bis-Tris gel in 1X MOPS-SDS running buffer for 50 min at 180 V. Complexes were transferred to nitrocellulose for 1.5 h at 30 V before being visualized on a LI-COR Odyssey infrared imager. The fluorescent signal from adaptorized hnRNP A1 and bound RNA^125^ was quantified using Image Studio Lite. The nitrocellulose membrane was subsequently blotted with mouse anti-hnRNP A1 antibody, clone 4B10 (Millipore Canada) at 1:1000 and donkey anti-mouse IR680 antibody at 1:10,000 to confirm successful immunoprecipitation.

### Identification of putative hnRNP A1 binding sites

Sequencing reads were extracted from each run and adapter trimmed using bcl2fastq (version 2.19.0.316; Illumina) with the following settings: “–use-bases-mask Y*,I8Y*,Y* --minimum-trimmed-read-length 0 –mask-short-adapter-reads 0”. Residual sequencing adapters and low-quality bases were trimmed using fastp^115^ with default settings except for “-f 5 -Y 0 -g” and the adapter sequences specified by PerkinElmer for use with the Bioo NEXTFlex v3 kit. For peak calling methods, adapter-trimmed reads were aligned to the GRCm38 mouse reference genome using STAR v2.5.1b^116^. The first four bases of each read were used as a UMI and deduplicated using NuDup v2.3. Cluster identification was performed using CLIPper^47,126^ for individual samples and PEAKachu^48^ for biological replicates in Galaxy^127^. Tools from Galaxy CLIP-Explorer^128^, including MEME suite^129^ and RNA-centric annotation system (RCAS)^58^, were used for motif and genic region binding, respectively. Genic region proportions were determined based on clusters that mapped to annotated regions. For assembly based data analyses, adapter-trimmed reads were assembled into contigs using rnaSPAdes^50^ (from SPAdes, version 3.15.3) with default settings and aligned to the GRCm38 mouse reference genome using minimap2^130^ (version 2.17) in spliced alignment mode to identify regions corresponding to putative hnRNP A1 RNA binding sites. Sites identified in the matched input or pooled IgG control data were considered to be background signal and were depleted from the set of sites for each immunoprecipitation sample using bedtools *subtract*^131^ (version 2.29.2) with default settings. Overlapping and book-ended sites were combined using bedtools *merge* with default settings for each sample to generate an hnRNP A1 footprint. Footprints that were consistent between the majority of samples in each condition (2/3 or 2/4 samples with >1nt overlap) were considered high quality and were carried forward for downstream analysis as the gene names for the closest feature on the reference genome. Data was visualized in IGV and LiftOver was used to convert genomic coordinates between the human GRCh38 and mouse GRCm38 reference genomes using UCSC Genome Browser.

#### IDR analysis

Merging of peak clusters from CLIPper was performed using the merge_peaks program as specified in the ENCODE eCLIP-seq Processing Pipeline v2.2^49^. This pipeline uses a python-based command line tool IDR to calculate reproducibility from merged peak clusters, however, certain aspects of the IDR program implementation and its integration into the merge_peaks pipeline were incompatible with the EAE data. Therefore, the generation of IDR scores and the calculation of the rescue and self-consistency ratios was performed with custom code in python using the packages seaborn and matplot lib as well as several of the functions from the IDR’s source code. A limitation of this approach is that it can only be utilized for two replicates. Therefore, all pairwise comparisons were individually performed within a group but limits claims that can be made to evaluating individual replicate reproducibility as opposed to entire group reproducibility. The majority of comparisons made for the EAE datasets were considered acceptable.

### Western blotting

#### CLIPseq samples

Input samples were precipitated by adding 7 volumes of acetone and chilling at −20 °C for 20 min. Samples were then pelleted at 18,000 × *g* for 20 min at 4 °C, acetone was removed, and then both immunoprecipitated protein samples were boiled in 2X SDS lysis buffer with freshly added 5% β-mercaptoethanol for 10 min at 95 °C. Samples were then magnetized to deplete them of magnetic beads and the supernatants were run on a 10% denaturing Tris-glycine acrylamide gel (standard SDS-PAGE) with a Licor Chameleon Due Pre-Stained Protein ladder (Licor; Cedarlane Labs). Protein was transferred to PVDF membranes using the semi-dry transfer method and a Bio-Rad TransBlot Turbo at 10 V for 40 min. Membranes were dried overnight, then rehydrated in methanol and blocked for 1 h with 5% goat serum (Jackson Immunoresearch Labs; Cedarlane Labs) in Tris-buffered saline (TBS). Membranes were serially probed in TBS + 0.1% Tween-20: first, with mouse anti-hnRNP A1 antibody, clone 4B10 (Millipore Canada) at 1:1000 and goat anti-mouse IgG (H + L)-HRP-conjugated secondary antibody (Bio-Rad Canada) at 1:3000 in TBS-T, then imaged; and second, with mouse anti-β-actin antibody, clone 8H10D10 (Cell Signaling Technology) at 1:1000 and goat anti-mouse HRP-conjugated secondary antibody, then imaged. HRP signal was visualized using Clarity Western ECL Substrate (Bio-Rad Canada) according to the manufacturer’s protocol, detected using a Bio-Rad Chemidoc, and images were prepared using Image Lab v6.1 software (Bio-Rad Canada).

#### Human, mouse, and cell culture samples

Fresh frozen human gray matter tissue was processed using a Tissue Tearor homogenizer in 5 volumes of T-PER tissue protein extraction reagent (Fisher Scientific Canada) containing protease inhibitors (Millipore Sigma 04693159001). Lysates were centrifuged at 10,000 × *g* for 5 min and supernatants collected and stored at −80 °C until further use. Mouse spinal cords were processed identically to CLIPseq input samples without a UV-crosslinking step. Neuro2A samples were harvested using CytoBuster Protein Extraction Reagent (Fisher Scientific Canada 710093MI) containing protease inhibitors as per the manufacturer’s protocol. For all types of samples, 40 μg of protein per sample was precipitated in 5 volumes of acetone and chilled at −20 °C for 20 min. Samples were then pelleted at 18,000 × *g* for 20 min at 4 °C, acetone was removed, and samples were boiled in 2X SDS lysis buffer with freshly added 5% β-mercaptoethanol for 10 min at 95 °C. Samples were run via SDS-PAGE, transferred to PVDF membrane, and blotted as described above. Membranes were incubated with primary antibody, including mouse anti-hnRNP A1 antibody, clone 4B10 (1:1000), rabbit anti-Abi2 (ProteinTech; 1:2000), mouse anti-β-actin antibody, clone 8H10D10 (Cell Signaling Technology; 1:1000) or rabbit anti-β-actin antibody (Cell Signaling Technology; 1:1000) followed by incubation with goat anti-mouse IgG (H + L)-HRP-conjugated or goat anti-rabbit IgG (H + L)-HRP-conjugated secondary antibody at 1:3000 then imaged. Western blots were quantified using band densitometry via ImageJ.

### Primary neuron culture

#### Primary neuron generation

Pregnant female C57Bl/6 mice were obtained from Charles River. Embryos staged at approximately E14-16 were used for experiments. Cortical tissue was isolated from the mouse embryos and collected in ice cold Earle’s balanced salt solution. Isolation of neurons was performed by using the Papain Dissociation System (Worthington Biochemical Corporation: LK003150), following the manufacturer’s protocol. Primary neurons were plated at a density of 1.5 × 10^6^ cells per mL onto sterile poly-d-lysine coated glass coverslips or wells in 6- or 24-well plates. Primary neuron cultures were maintained in Neurobasal A media supplemented with 2% B-27, 2 μM L-glutamine and 0.5% penicillin-streptomycin. Primary neurons were transduced as described below after 2 days in culture.

#### Immunofluorescence of primary mouse embryonic neurons

Cells were fixed and immunostained for imaging analysis at 72 h post-transduction and prepared as described in Libner et al.^46^. Briefly, neurons were fixed in 3.7% formaldehyde diluted in complete media for 15 min at 37 °C. Neurons were washed and blocked in 100% Seablock Blocking Buffer (ThermoFisher Scientific) for 1 h followed by incubation with chicken anti-beta-tubulin III antibody (Aves Labs) overnight at 4 °C. Following washes, neurons were incubated with anti-chicken IgY FITC (Aves Labs) secondary antibody for 1 h at room temperature. Coverslips were mounted onto slides using ProLong Gold Antifade Mountant with DAPI (Invitrogen).

### AAV generation

#### Cloning

All cloning was carried out using restriction endonucleases, buffers, and reagents from New England Biolabs Canada. PCRs were carried out using Q5 High Fidelity Hot-Start 2X Master Mix, and DNA assembly was carried out using NEBuilder HiFi DNA Assembly Master Mix. Primers are indicated in Supplementary Table 5, and plasmid details are found in Supplementary Table 6. pAAV-hSyn-mCherry was cloned by PCR and HiFi assembly. In brief, mCherry was PCR amplified from plasmid N-pmCry2PHR-A1(WT)-mCherry-C with primers **hSyn-Kpn-KOZAK-mCh-F** and **AAV-HindIII-mCh-R** and inserted into pAAV-hSyn-mScarlet (Addgene plasmid #131001; a gift from Karl Deisseroth) digested with BamHI and HindIII, to produce pAAV-hSyn-mCherry. pAAV-hSyn-hnRNPA1(WT)-mCherry was generated by PCR amplification from N-pmCry2PHR-A1(WT)-mCherry-C with primers **hSyn-Kpn-A1-F** and **AAV-HindIII-mCh-R**, and inserted into pAAV-hSyn-mScarlet digested with BamHI and HindIII. pAAV-hSyn-hnRNPA1(F263S)-mCherry was generated with two overlapping PCRs from N-pmCry2PHR-A1(WT)-mCherry-C (**hSyn-Kpn-A1-F** + **F263S-R**, and **F263S-F** + **AAV-HindIII-mCh-R**) and inserted into pAAV-hSyn-mScarlet digested with BamHI and HindIII. HiFi reactions were transformed into NEB Stable cells per manufacturer’s protocol and cultured on LB-Agar with ampicillin at 30 °C to ensure AAV genome stability. Colonies were screened by miniprep (Purelink Quick Plasmid Miniprep kit; ThermoFisher Scientific Canada) and Sanger sequencing. Sequence-verified plasmids used for virus rescue were prepared at Maxi- or Midi-prep scale (Purelink HiPure Plasmid Midiprep and Maxiprep kits; ThermoFisher Scientific Canada).

#### Cell culture

HEK293T cells (ATCC CRL-3216) were maintained in culture medium: DMEM (Corning; Fisher Scientific Canada) + 10% FBS (Gibco; Fisher Scientific Canada), and passaged by washing with Dulbecco’s PBS (D-PBS; Millipore Sigma Canada) and treating with trypsin (Sigma Aldrich Canada). Cells were incubated at 37 °C with 5% CO_2_, and discarded after 35 passages.

#### AAV production

All reagents were purchased from Fisher Scientific Canada unless otherwise noted. Adeno-associated virus (AAV) vector production was derived from the protocol published by Kimura et al.^132^, which contains recipes for all indicated buffers and ratios for all plasmid transfections, and was modified as follows: Pen/Strep was not used in the culture medium, aqueous two-phase partitioning was bypassed, and preparations did not require buffer exchange after ultracentrifugation. Viruses were quantified as genome copies per mL (gc/mL) by qPCR as described in Kimura et al.^132^ with ITR-qPCR-F and ITR-qPCR-R (Supplementary Table 5).

#### Transduction

Purified AAVs were diluted in primary neuron culture medium and added to cells by half-medium exchange to a final concentration of 1.5 ×10^11^ gc/mL (approx. 2.75 ×10^11^ gc per well in a 6-well plate for RNA extraction, or 5.2 ×10^10^ gc per well in a 24-well plate with glass coverslip for imaging). Transduction was permitted to occur overnight at 37 °C, then half the medium was again exchanged for fresh media. Cells were collected or fixed 72 h post-transduction.

### CRISPR

#### Cloning

**pU6-(BbsI)_CBh-Cas9-T2A-mCherry-H1-(BamHI)** (Addgene plasmid #64217; a gift from Ralf Kuehn) was digested with EcoRI and NheI (New England Biolabs Canada) to remove the mCherry fluorescent marker. The puromycin resistance gene (PuroR) was PCR amplified from **AAVS1_Puro_Tet3G_3xFLAG_Twin_Strep** (Addgene plasmid #92099; a gift from Yannick Doyon) using the forward primer **ggaggagaatcccggccctgctagcaccgagtacaagccc** and reverse primer **gctgatcagcgagctctaggaattctcaggcaccgggcttgc** (Integrated DNA Technologies), then inserted in place of mCherry to produce **pU6-(BbsI)_CBh-Cas9-T2A-PuroR-H1-(BamHI)** using the Gibson Assembly Cloning Kit (New England Biolabs Canada). The Gibson reaction was transformed into NEB Stable cells (New England Biolabs Canada) Clones were miniprepped using the Purelink Quick Plasmid Miniprep Kit (Thermo Fisher Scientific Canada) and verified by Sanger sequencing. gRNA primers (**CACCGGGAACACTAACAGACTGTG** and **AAACCACAGTCTGTTAGTGTTCCC**) were phosphorylated with T4 polynucleotide kinase (New England Biolabs Canada), and annealed by boiling, then reducing temperature 0.1 °C/second to 25 °C. In parallel, **pU6-(BbsI)_CBh-Cas9-T2A-PuroR-H1-(BamHI)** was digested with BbsI (New England Biolabs Canada) and gel purified, and the annealed primers were ligated into the vector with T4 DNA ligase (New England Biolabs Canada) to generate **pU6-(gA1-03)_CBh-Cas9-T2A-PuroR-H1-(BamHI)**. The ligation reaction was transformed into NEB Stable cells, clones were miniprepped and verified by Sanger sequencing, and then the selected plasmid clone was prepared for transfection using the Purelink HiPure Plasmid MidiPrep kit (Thermo Fisher Scientific, Canada).

#### Cell culture

Both Neuro2A cells (ATCC CCL-131), a mouse neuroblastoma cell line, and SK-N-SH cells (ATCC HTB-11), a human neuroblastoma cell line, were maintained in DMEM with 10% FBS and 1% penicillin/streptomycin (HyClone; Fisher Scientific Canada) in an incubator at 37 °C with 5% CO_2_ and discarded after 35 passages.

#### Transfection and short-term selection

Neuro2A cells were plated at a density of 350,000 cells per well of a 6 well plate. 24 h after plating, cells were transfected with 2.5 μg of pU6-(gA1-03)CBh-Cas9-T2A-PuroR-H1-(BamHI) plasmid and Lipofectamine 2000 (Invitrogen; Fisher Scientific Canada) per well or Lipofectamine 2000 alone. After overnight incubation, media was changed to include puromycin (5 μg/mL) to select for plasmid-positive cells in transfected wells. Following 3 days of incubation with puromycin, media was changed, and cells were allowed to recover for 24 h before being used for downstream analyses.

#### Cycloheximide treatment

SK-N-SH cells were plated at a density of 700,000 cells per well of a 6 well plate. 24 h after plating, cells were treated with cycloheximide (CHX; Cell Signaling Technology) at 50 μg/mL or an equivalent volume of DMSO for 4 h.

### Total RNA extraction for RT-PCR

#### Spinal cord lysates

Samples were subjected to phenol/chloroform/isoamyl alcohol purification and RNA cleanup as described above and eluted into nuclease-free water. Samples were then treated with Turbo Dnase and subjected to a second round of RNA cleanup using the Zymo RNA Clean & Concentrator Kit as described above, then quantified by Qubit RNA High Sensitivity Assay (Invitrogen; Fisher Scientific Canada).

#### Human brain samples, primary neurons, and cell lines

RNA was isolated using the Rneasy Plus Universal Mini Kit (Qiagen), which included a gDNA depletion step. RNA was eluted in water and quantified using a Nanodrop 1000 spectrophotometer (ThermoFisher).

### RT-PCR

#### Gene-specific RT-PCR

1 μg (human brain) or 400 ng (mouse spinal cord, primary neurons, and cell lines) total RNA was subjected to RT-PCR using ProtoScript II Reverse Transcriptase (New England Biolabs Canada) and its recommended protocol. Species-appropriate gene-specific primers (Supplementary Table 5) were pooled as indicated and used at a final pool concentration of 2 μM. NRT (no reverse transcriptase) samples were prepared identically, but reverse transcriptase was replaced with nuclease-free water.

#### Total RNA RT-PCR

500 ng of total RNA was subjected to RT-PCR with a combination of random-hexamer and oligo-dT primers via the QuantiTect Reverse Transcription Kit (Qiagen Canada) according to the manufacturer’s protocol. NRT samples were prepared identically, but reverse transcriptase was replaced with nuclease-free water.

#### qPCR

Total RNA RT-PCR was diluted 1/20 into a qPCR mastermix containing the indicated primers (Supplementary Table 5) at 250 nM each, 2X PowerUp SYBR Green Master Mix (Applied Biosystems; Fisher Scientific Canada), and nuclease-free water to a final volume of 20 μL. Samples and matched NRT controls were run on a QuantStudio 3 qPCR system according to the PowerUp protocol, and Ct values were determined using the QuantStudio Design & Analysis Software (v1.5.1). Fold change was determined using ΔΔCt analysis.

#### Amplicon PCR

Gene-specific RT-PCR products were diluted 1/625 into a reaction with Q5 Hot Start High-Fidelity 2X Master Mix (New England Biolabs Canada) and the indicated primers (Supplementary Table 5) at 400 nM each. PCRs were then carried out in an Axygen Maxygene II thermocycler according to the recommended Q5 manufacturer’s protocol: 98 °C for 30 seconds; 35 cycles of 98 °C for 10 seconds, 54-66 °C for 30 seconds, and 72 °C for 30 seconds; then 72 °C for 2 min. Samples were resolved along with the 1 kb DNA Plus Ladder (Invitrogen; Fisher Scientific Canada) on a 2% agarose gel in Tris-Borate-EDTA buffer (TBE) poured with 1/10,000 dilution of SYBR Safe DNA Gel Stain (Invitrogen; Fisher Scientific Canada) and visualized using an E-Gel UV Imager and the associated GelCapture Imaging Software (Invitrogen; Fisher Scientific). Band densitometry was carried out using Fiji ImageJ 1.53q^133^.

### RNA EMSA

#### Cloning and recombinant protein production

The plasmids N-MBP-TEV-A1WT-C (MBP: maltose binding protein; A1WT: wild-type A1) and N-MBP-C were previously cloned using HiFi DNA assembly cloning (NEBuilder HiFi DNA Assembly Cloning Kit, New England BioLabs)^134^. For production and purification of recombinant proteins, plasmids were first transformed in BL21 (DE3) *E. coli* and were grown overnight on agar plates at 37 °C with kanamycin (Kan). Multiple colonies were selected and grown overnight with shaking (300 rpm) in 5 mL Terrific Broth supplemented with 5% glycerol (TBG) and Kan. Cultures were harvested (6800 × *g* for 5 min) and re-suspended in 1.0 mL of TGB with Kan. 100 µL of resuspension was added to 50 mL of TGB with Kan and incubated at 37 °C with shaking (300 rpm) for ~2–3 h until a spectrophotometer OD_600_ reading of 0.3-0.4 was obtained. Cultures were then harvested (6800 × *g* for 5 min) and resuspended in 5.0 mL of TGB with Kan, and then added to 250 mL of TGB with Kan. Antifoam B emulsion was also added (0.008%, v/v) to prevent foam formation. Cultures were incubated at 37 °C with shaking (300 rpm) for ~4–5 h until a spectrophotometer OD_600_ reading of ~1.0 was obtained. A new dose of Kan was then added, along with Isopropyl-β-D-Thiogalactopyranoside (IPTG) at a final concentration of 1.0 mM. Cultures were incubated at 37 °C with shaking (300 rpm) for 4 h. Protein isolation was then performed by harvesting the final culture (6800 × *g* for 5 min) and lysing the cells with 7 mL lysis buffer [700 µL CelLytic B, one cOmplete Mini, EDTA-free protease inhibitor cocktail tablet, 1.0 mM Dithiothreitol (DTT) and 10% Glycerol in 0.01 M Phosphate Buffer Saline (PBS), pH 7.4]. Cell lysates were then freeze/thawed (−80 °C, 5 min/37 °C, 2 min) three times before centrifugation at 18,000 × *g* for 15 min at 4 °C. Supernatants were then run through Cytiva MBPTrap HP pre-packed columns, according to the manufacturer’s instructions. Columns were then washed with Binding Buffer (200 mM NaCl, 1.0 mM EDTA, 1.0 mM DTT, 10% Glycerol in 20 mM Tris-HCl, pH 7.4) and protein was eluted with Elution Buffer [200 mM NaCl, 1.0 mM EDTA, 1.0 mM DTT, 10% Glycerol, 10 mM D-(+)-Maltose in 20 mM Tris-HCl, pH 7.4]. Collected protein eluates were concentrated and buffer exchanged (protein buffer: 150 mM NaCl in 10 mM Sodium Phosphate Buffer, pH 6.8) using Amicon Ultra-2 3 kDa centrifugal filter units, according to the manufacturer’s instructions. These were then snap frozen in 200 µL aliquots in liquid nitrogen and stored at −80 °C until use.

#### EMSA

hMACF1 RNA was synthesized by IDT with a 2′-O-methylation (2′-OME) on the 3′ end of the RNA sequence (Supplementary Table 5). Label-free RNA EMSA was performed according to Seo et al.^135^. with modifications. Briefly, a 5% native TBE polyacrylamide gel was poured. After polymerization, the gel was pre-run in 0.5X TBE at 60 V for 1 h. During this time, recombinant protein and hMACF1 RNA were combined in 1X binding buffer and incubated for 30 min at room temperature. 500 nM RNA was incubated with increasing amounts of recombinant protein (0.04-5 μg): either MBP alone (N-MBP-C) or MBP-A1 WT (N-MBP-TEV-A1WT-C). After incubation, 2 μL of 25 mg/mL Ficoll solution was added to each tube and 20 μL of recombinant protein:RNA was loaded onto the gel. Gels were run at 60 V for 1.5 h after which they were incubated with SYBR Gold Nucleic Acid Gel Stain (Invitrogen) diluted in 0.5X TBE for 15 min while shaking. Gels were imaged using a Bio-Rad Chemidoc.

### Neurite quantification

For neurite quantification, primary neuron cultures were stained for beta-tubulin III to outline neurites. 20 random fields of view were acquired for each replicate using a Plan Apochromat 40X Oil objective, with a 1.40 numerical aperture, with identical acquisition settings on a Zeiss Axio Observer 7, inverted, fluorescent microscope (Carl Zeiss Microscopy, LLC) and exported as original TIFFs using Zeiss ZEN 3.1 Blue Edition software (Carl Zeiss Microscopy, LLC). NeuriteTracer, an ImageJ plugin, was used to trace and measure neurites and count nuclei^136^. The average neurite length for individual images was determined by dividing the total neurite length in an image by the total number of nuclei. To account for differences in neurite outgrowth between biological replicates, neurite length was normalized to hnRNP A1(WT)-transduced neurons within each replicate. Therefore, results are reported as a percentage of neurite length compared to hnRNP A1(WT).

### Statistical analysis

All statistical analyses, including statistical tests and graph generation, were performed using GraphPad Prism v. 8, unless otherwise indicated. For all analyses, *p* < 0.05 was considered statistically significant. Non-parametric tests were used for data that was not normally distributed.

### Reporting summary

Further information on research design is available in the Nature Portfolio Reporting Summary linked to this article.

### Supplementary information

Supplementary Information

Description of Additional Supplementary Files

Supplementary Data 1-4

Reporting Summary

### Source data

Source Data

## Data Availability

All data generated or analyzed during this study are included in this article and its Supplementary Information files. All requests for raw data and materials should be addressed to the corresponding author. Source data are provided with this paper. RNA sequencing data have been deposited in NCBI’s Gene Expression Omnibus and are accessible through GEO Series accession number GSE207680 (human RNAseq) and GSE208093 (mouse CLIPseq). Source data are provided with this paper.

## Code availability

The ENCODE eCLIP-seq Processing Pipeline v.2.2 was used for CLIPseq IDR data analysis^137^.
