# Supplementary material for: hnRNP A1 dysfunction alters RNA splicing and drives neurodegeneration in multiple sclerosis (MS)
Source: Nat Commun. 2024 Jan 8;15:356. doi: 10.1038/s41467-023-44658-1 (PMC10774274; doi:10.1038/s41467-023-44658-1)
Supplement: Supplementary file 1 — Supplementary Information [file 41467_2023_44658_MOESM1_ESM.pdf]

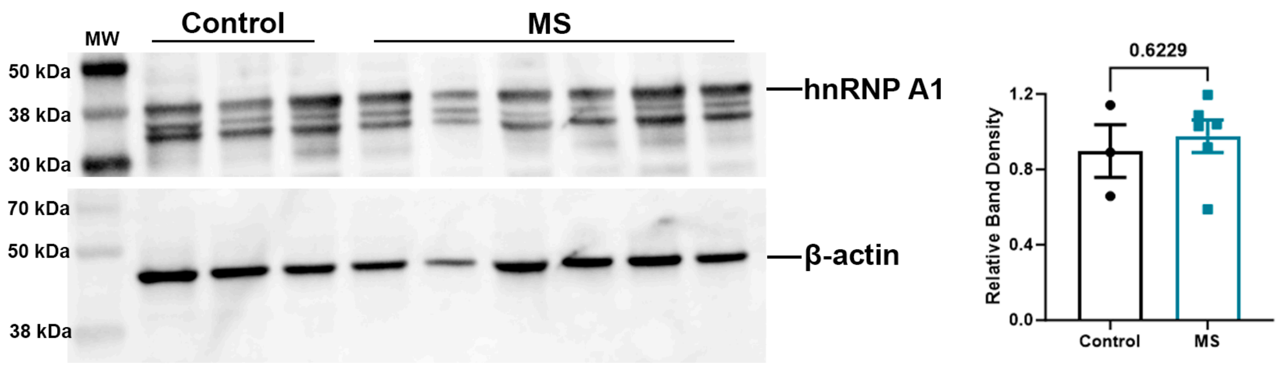

Supplementary Figure 1 Total hnRNP A1 protein levels do not differ between control and MS cortex.
Protein isolated from control (n=3) or MS (n=6) human grey matter samples was assayed by western blot (left) for hnRNP A1 expression, normalized to β-actin. Band densitometry (right) confirms that there is no significant difference in hnRNP A1 protein levels between control and MS samples. Values derived from two separate technical replicates with the same biological replicates. Data are plotted as the mean +/- SEM. Unpaired t-test, two tailed.

Supplementary Table 1

Human sequencing QC information

Sample	Library ID	DNA [ng/uL]	Fragment size	# read pairs	# read pairs aligned	% duplicates	# unique read pairs	# read pairs overlapping an exon	% unique pairs used for expression
C1	R2100154	14.2	298	16904102	15089081	0.0603	14179929	7431942	52.412
C3	R2100156	10	301	15540237	13803916	0.0507	13103499	6177234	47.142
MS1	R2100157	17.4	294	14354961	12277806	0.0693	11426356	5698115	49.868
MS2	R2100162	7.1	305	22768732	19615526	0.0607	18424834	8541250	46.357
MS3	R2100163	6.3	283	9149373	6872488	0.0701	6390576	4125301	64.553
C2	R2100164	19.8	318	17715767	15629625	0.2335	11979428	5907386	49.313

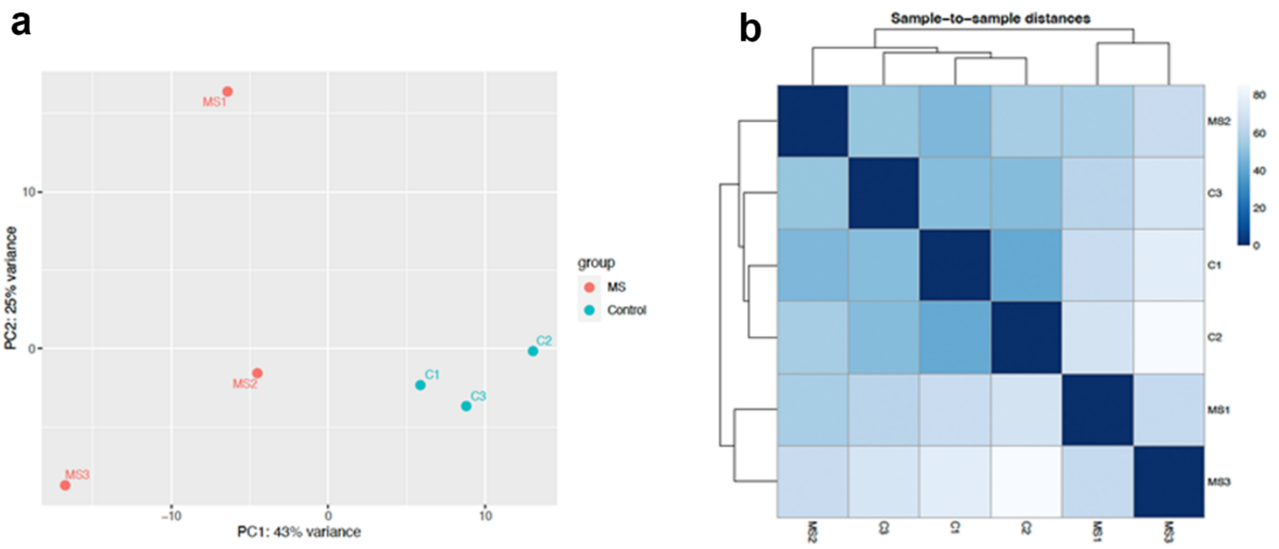

Supplementary Figure 2. Control and MS brain samples cluster separately after RNAseq.
PCA plot (a) and sample-to-sample distance plot (b) demonstrate separation of samples based on condition (MS vs. control).

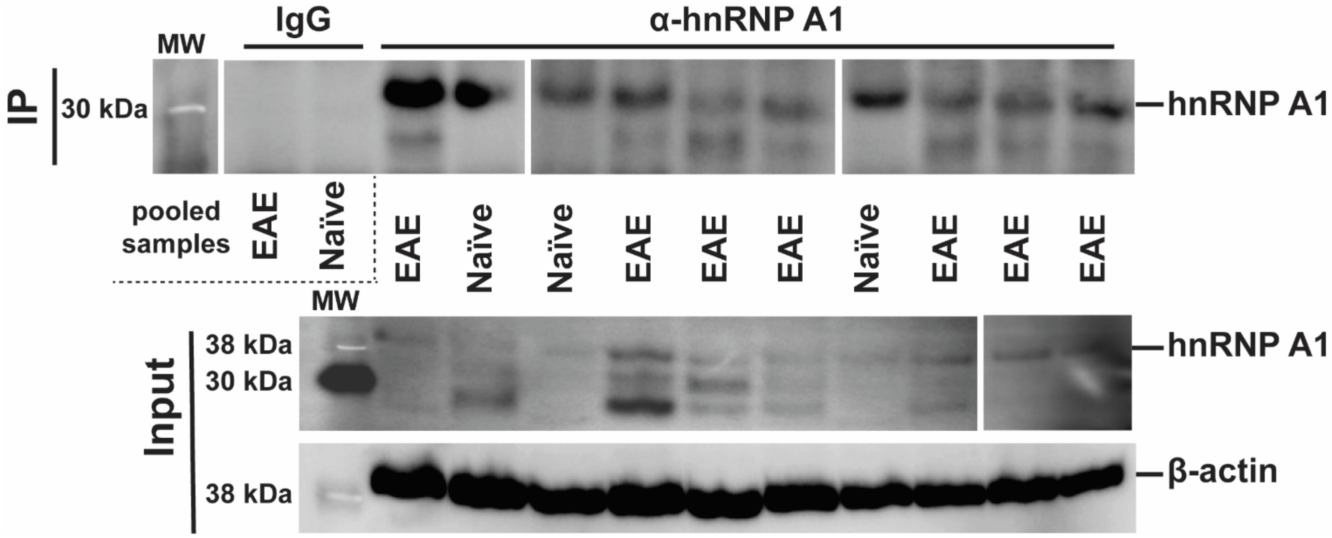

Supplementary Figure 3. Immunoprecipitation of hnRNP A1 from naïve and EAE mice. Western blot for hnRNP A1 of immunoprecipitation and input samples, illustrating successful immunoprecipitation for hnRNP A1 in CLIP samples. As a control for specificity, naïve and EAE samples were pooled separately and immunoprecipitated with IgG, which showed no reactivity for hnRNP A1.

Supplementary Table 2

CLIPseq sequencing QC information

Sample	EAE Score	Library ID	Mouse Designation	# read pairs	Average fragment size (bp)	[DNA] (ng/uL)	Mapped Unique Reads
EAEIP_1	2.5	R2100080	Severe EAE 1	10711958	193	1.12	5353320
EAEIP_3	1.5	R2100081	Mild EAE 1	7171960	201	1.72	5647679
NaiveIP_1	0	R2100082	Naïve 1	6798878	206	2.32	5497133
EAEIP_5	2.5	R2100083	Severe EAE 4	11001563	209	2.72	10909456
NaiveIP_2	0	R2100084	Naïve 2	11777138	206	5.18	2344707
EAEIP_7	1	R2100085	Mild EAE 3	8274902	204	1.21	8240409
NaiveIP_3	0	R2100086	Naïve 3	6189535	205	0.91	5280237
EAEIP_2	3	R2100087	Severe EAE 2	26320198	356	1.48	6910620
EAEIP_4	1.5	R2100088	Mild EAE 2	8928593	202	2.14	5707252
EAEIP_6	3	R2100089	Severe EAE 3	9505028	189	2.78	5117809
EAEIgG_1		R2100090		7402768	187	3.08	2984897
NaiveIgG_1		R2100091		11044553	220	2.72	1248739
EAEinput_1		R2100092		13616972	172	1.96	6166885
EAEinput_3		R2100093		15911829	169	2.56	2654223
Naiveinput_1		R2100094		9959143	177	0.79	3868549
EAEinput_5		R2100095		9696735	170	0.88	3237136
Naiveinput_2		R2100096		53819781	171	2.54	9964993
EAEinput_7		R2100097		13158238	173	1.85	6283946
Naiveinput_3		R2100098		11437231	172	2	5583952
EAEinput_2		R2100099		67985768	173	4.84	19082713
EAEinput_4		R2100101		13467503	166	4.3	5319815
EAEinput_6		R2100102		14875683	169	5.84	7726751

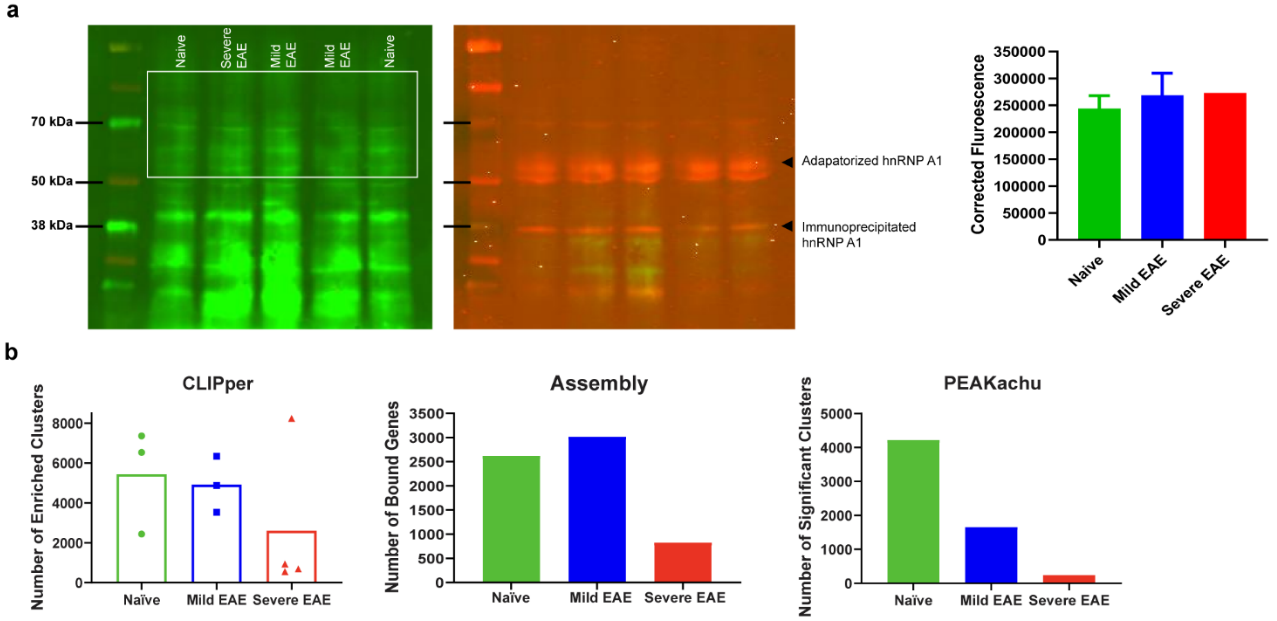

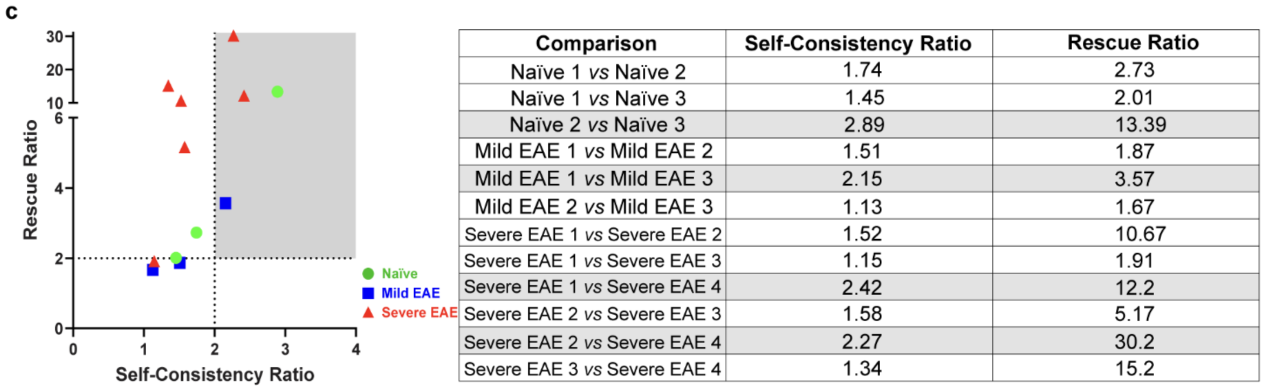

Comparison	Self-Consistency Ratio	Rescue Ratio
Naïve 1 *vs* Naïve 2	1.74	2.73
Naïve 1 *vs* Naïve 3	1.45	2.01
Naïve 2 *vs* Naïve 3	2.89	13.39
Mild EAE 1 *vs* Mild EAE 2	1.51	1.87
Mild EAE 1 *vs* Mild EAE 3	2.15	3.57
Mild EAE 2 *vs* Mild EAE 3	1.13	1.67
Severe EAE 1 *vs* Severe EAE 2	1.52	10.67
Severe EAE 1 *vs* Severe EAE 3	1.15	1.91
Severe EAE 1 *vs* Severe EAE 4	2.42	12.2
Severe EAE 2 *vs* Severe EAE 3	1.58	5.17
Severe EAE 2 *vs* Severe EAE 4	2.27	30.2
Severe EAE 3 *vs* Severe EAE 4	1.34	15.2

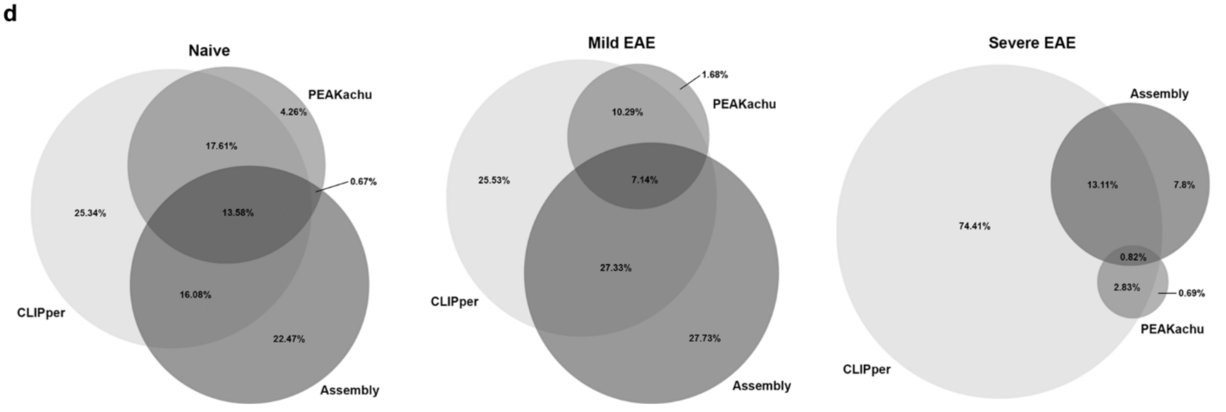

Supplementary Figure 4. hnRNP A1 from mice with severe EAE binds fewer distinct RNAs, validated across multiple methods of bioinformatic identification of targets.
(a) Selected samples from each group (naïve n=2, mild EAE n=2, severe EAE n=1) were used for irCLIP to examine hnRNP A1-bound RNA. Corrected fluorescence quantification demonstrates comparable amounts of RNA bound to hnRNP A1 compared across groups. (b) Comparison of the three methods used to identify hnRNP A1-bound RNAs. All methods demonstrated a significant loss of target binding in severe EAE compared to naïve and mild EAE. For CLIPper, data are plotted as the number of enriched clusters for individual animals (naïve n=3, mild EAE n=3, severe EAE n=4). For assembly and

PEAKachu, data are plotted as the average of the number of bound genes or number of significant clusters, respectively, for each group. (c) Conservative (high-confidence) IDR analysis of CLIPper peaks demonstrates reproducibility between individual samples within each group. Self-consistency (x-axis) and rescue (y-axis) ratios are presented, which are comparisons between two samples. Thus, each point indicates a comparison between two samples as indicated in the embedded table (severe EAE: red triangles; mild EAE: blue squares; naïve: green circles). Replicates are considered to be acceptable if at least one of the ratios is less than 2. The gray box region in the plot and grayed entries in the table indicate samples with both high self-consistency and rescue ratios, which together indicate comparisons of concern according to ENCODE guidelines. It is noteworthy that all comparisons of concern for severe EAE samples are comparisons against Severe EAE 4 (embedded table), which is also a visual outlier in all footprinting experiments. (d) Venn diagrams illustrating the overlap between the identified genes for each footprinting approach in naïve, mild EAE, and severe EAE. The majority of genes found to be bound by hnRNP A1 using PEAKachu were also identified using the CLIPper and assembly approaches.

Supplementary Table 3

Peak calling methods comparison

CLIPper			
**Animal**	**Group**	**# enriched clusters**	**# genes**
Naïve 1	Naïve	7366	2791
Naïve 2	Naïve	2443	1327
Naïve 3	Naïve	6537	2649
EAE 1	Severe EAE	688	455
EAE 2	Severe EAE	942	647
EAE 3	Mild EAE	6345	2621
EAE 4	Mild EAE	3529	1773
EAE 5	Severe EAE	8242	2941
EAE 6	Severe EAE	557	394
EAE 7	Mild EAE	4875	2224
**PEAKachu**			
	**Group**	**# significant clusters**	**# genes**
	Naïve	4213	1790
	Mild EAE	1653	937
	Severe EAE	239	166
**Assembly**			
	**Group**	**# genes**	
	Naïve	2617	
	Mild EAE	3018	
	Severe EAE	824	

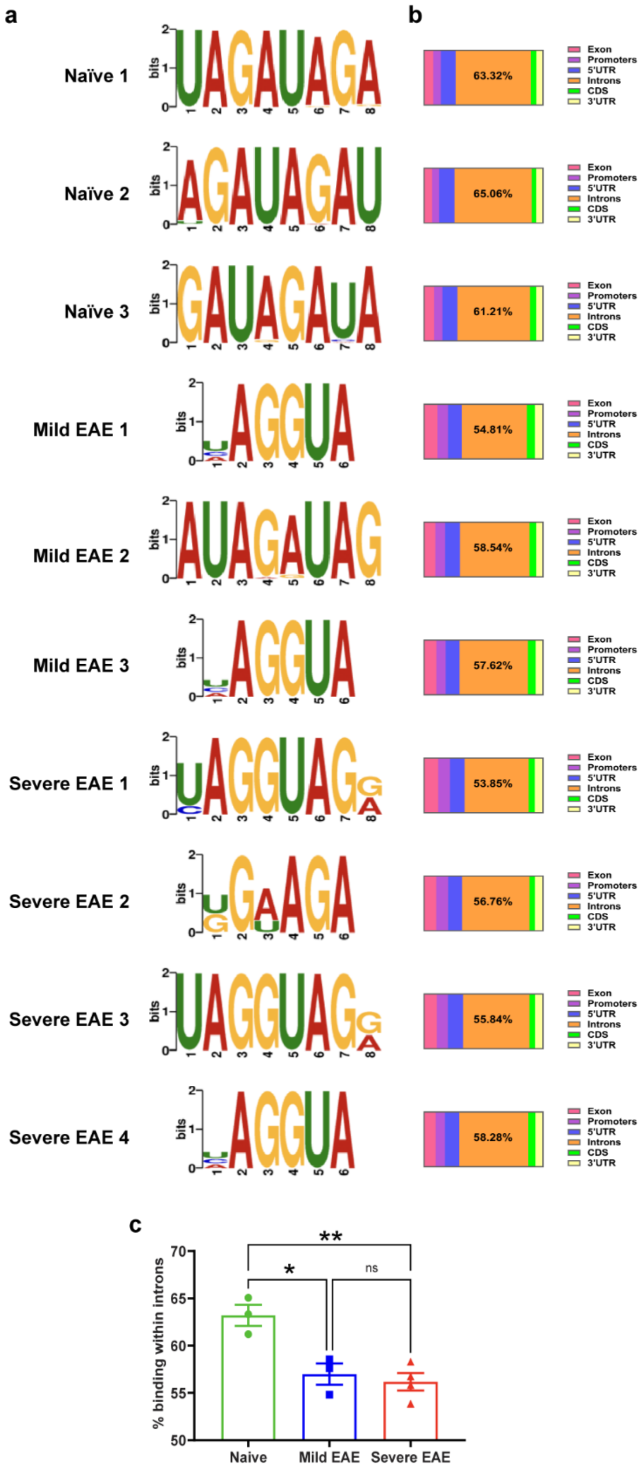

Supplementary Figure 5. hnRNP A1 changes sequence and intronic binding preferences in mice with EAE.

(a) Binding motif analysis demonstrates that hnRNP A1 binds with specificity to AG-rich regions with enrichment for a UAG motif in naïve mice and strong representation of an altered AGGU motif in EAE mice. (b) In all mice, hnRNP A1 predominantly binds introns. However, there is reduced intronic binding in EAE animals (c). One-way ANOVA with Tukey's post-hoc tests with $*p<0.05$ (exact $p=0.0047$), $**p<0.01$ (exact $p=0.0126$), ns=not significant. Data are plotted as mean +/- SEM for naïve (green circles, n=3) and EAE (mild, n=3 blue squares; severe, n=4 red triangles) mice.

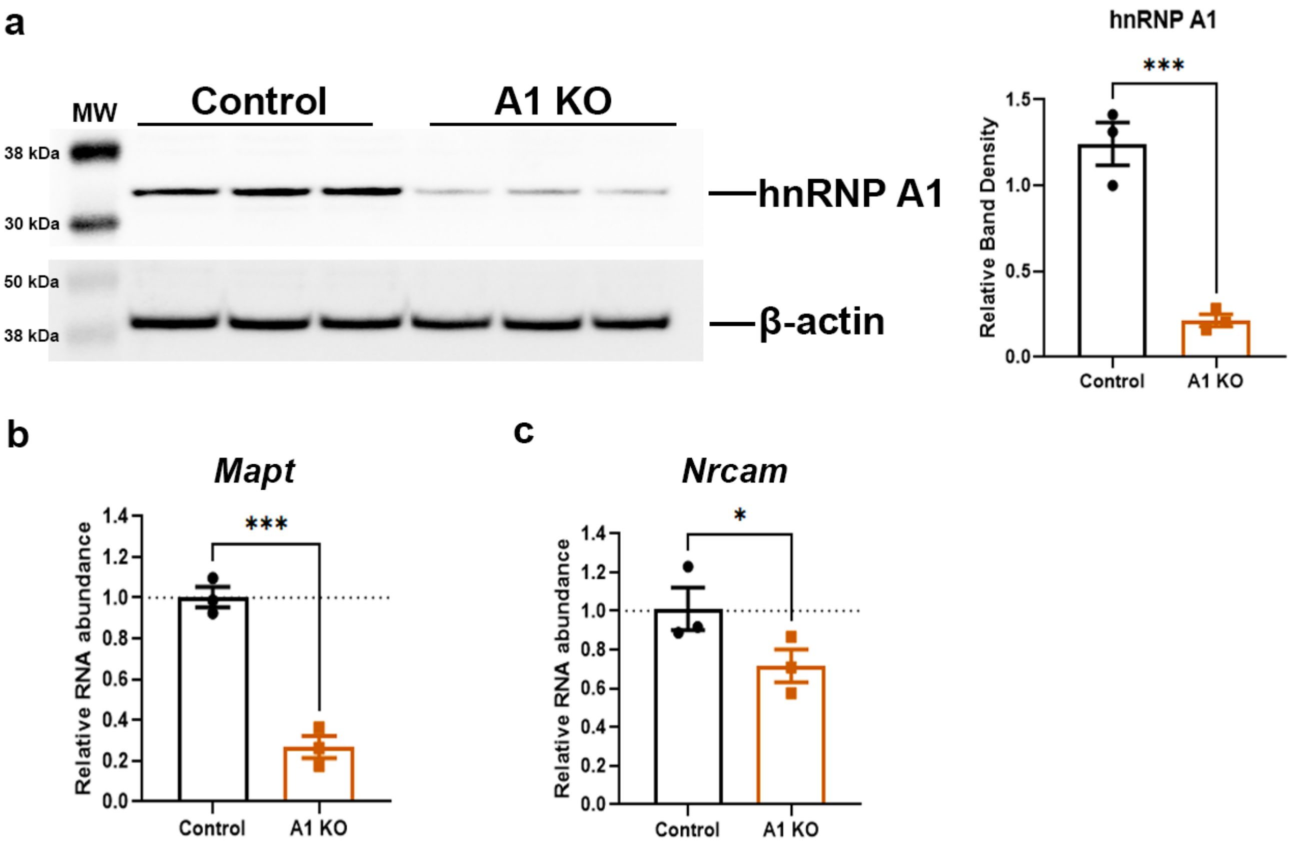

Supplementary Figure 6. Knockout of hnRNP A1 produces alterations in RNA abundance of known hnRNP A1 binding targets.
(a) CRISPR-mediated knockout of hnRNP A1 in Neuro2A cells was highly efficient as evidenced by western blot (n=3 biological replicates for control and A1 KO). (b, c) qPCR confirmed changes in RNA abundance of *Mapt* (b) and *Nrcam* (c) following hnRNP A1 knockout (n=3 biological replicates for control and A1 KO biological replicates). Unpaired t-test, one tailed with *p<0.05 (exact p=0.0007 for (a) and p=0.0500 for (c)) ***p<0.001 (exact p=0.0003). Data are plotted as the mean +/- SEM.

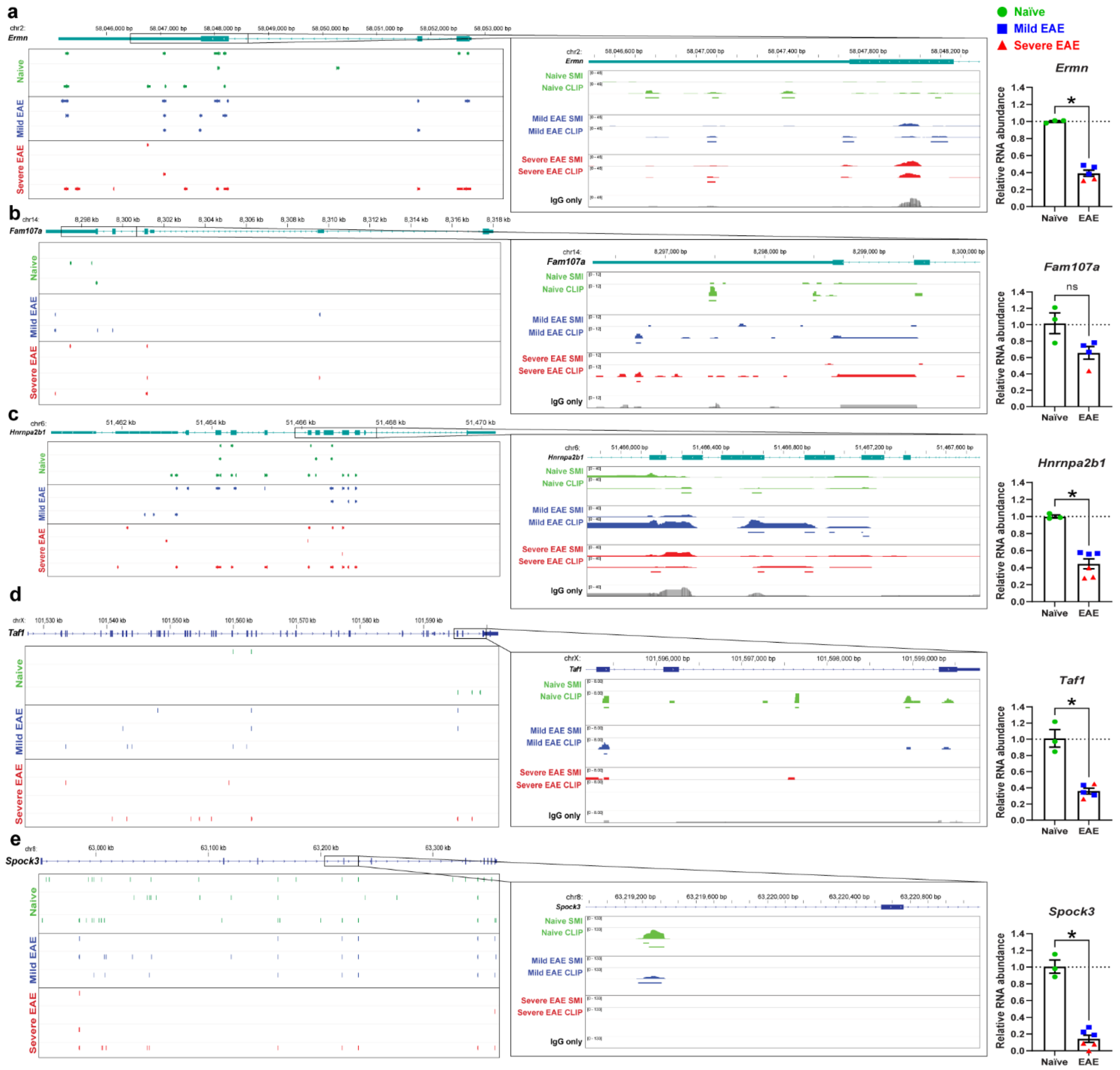

Supplementary Figure 7. Additional hnRNP A1-bound RNAs show altered abundance in mice with EAE.

Additional targets, including *Ermn* (a), *Fam107a* (b), *Hnrnpa2b1* (c), *Taf1* (d), and *Spock3* (e), show changes in hnRNP A1 binding and subsequent abundance at different disease stages. CLIPper-identified hnRNP A1 binding sites within targets (left) where inset images show read density in reads per million for naïve, mild EAE, and severe EAE CLIP samples with paired SMI and IgG-only controls. CLIPper identified peaks are indicates by boxes below each respective animal. qPCR (right) was used to examine changes in expression between naïve and EAE mice. Unpaired t-test, one-way with *p<0.05, ns=not significant. Data are plotted as mean +/- SEM for naïve (green circles, n=3 for all targets) and EAE (mild, n=3 blue squares; severe, n=1 for *Fam107a* n=2 for *Ermn*, *Taf1* n=3 for *Hnrnpa2b1*, *Spock3* red triangles) mice. Variation in severe EAE sample number is due to failure to detect the indicated transcript in some samples, although housekeeping and other genes were quantifiable in these samples.

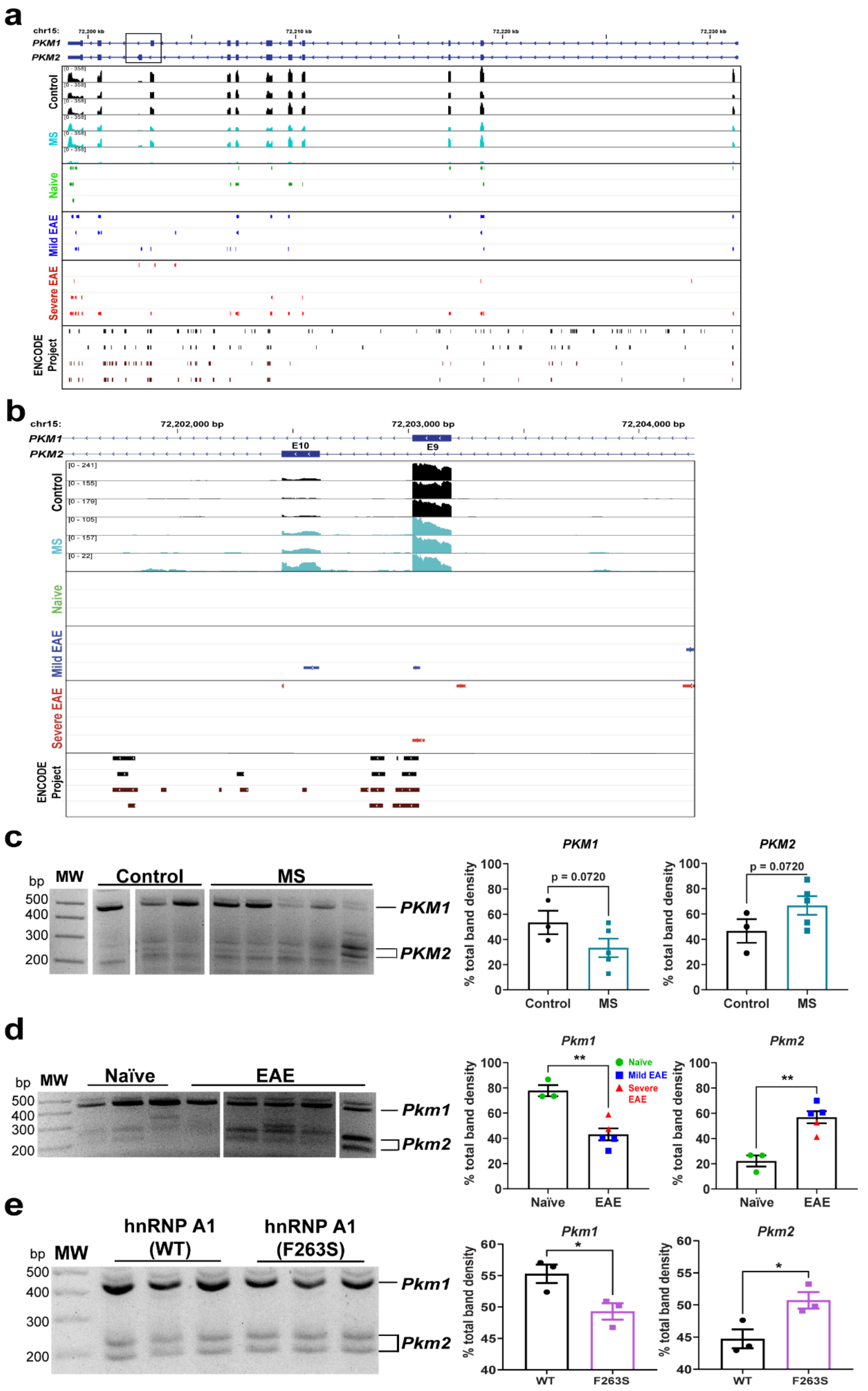

Supplementary Figure 8. hnRNP A1 dysfunction results in alternative splicing of *PKM/Pkm*.

(a) RNA sequencing reads in *PKM* from MS samples (light blue) as compared to controls (black). LiftOver was used to overlap mouse CLIPseq tracks of hnRNP A1 binding sites onto human *PKM*. Data of hnRNP A1 binding sites on human *PKM* from the ENCODE project were incorporated to examine binding sites from previously published datasets. Exons 9 and 10 are shown in the boxed in region of (a). (b) Zoomed-in IGV snapshot of the region from (a) where RNA sequencing reads in *PKM* show increased reads in exon 10 (E10) in MS samples (light blue) as compared to controls (black), indicative of *PKM2* enrichment. LiftOver was used to overlap mouse CLIPseq tracks of hnRNP A1 binding sites onto human *PKM*. Data of hnRNP A1 binding sites on human *PKM* from the ENCODE project were incorporated to examine binding sites from previously published datasets. (c-e) *PKM2/Pkm2* includes exon 10, which contains a PstI restriction endonuclease recognition site. For all experiments, to detect *PKM2/Pkm2* abundance, PCR amplification products were incubated with PstI before being visualized. The single top band is indicative of *PKM1/Pkm1* while the bottom doublet band is indicative of the digested *PKM2/Pkm2* isoform. Amplicon PCR and quantification demonstrate enrichment of the *PKM2* isoform in MS (n=5) as compared to control (n=3) in (c), enrichment of the *Pkm2* isoform in EAE spinal cord (mild, n=3 blue squares; severe, n=2 red triangles) compared to naïve (n=3 green circles) in (d), and enrichment of the *Pkm2* isoform in primary mouse neurons transduced with hnRNP A1(F263S) (n=3 biological replicates for WT and F263S) in (e). For all, two-tailed unpaired t-test with *$p<0.05$ (exact $p=0.0371$) and **$p<0.01$ (exact $p=0.0028$). Data are plotted as mean +/- SEM.

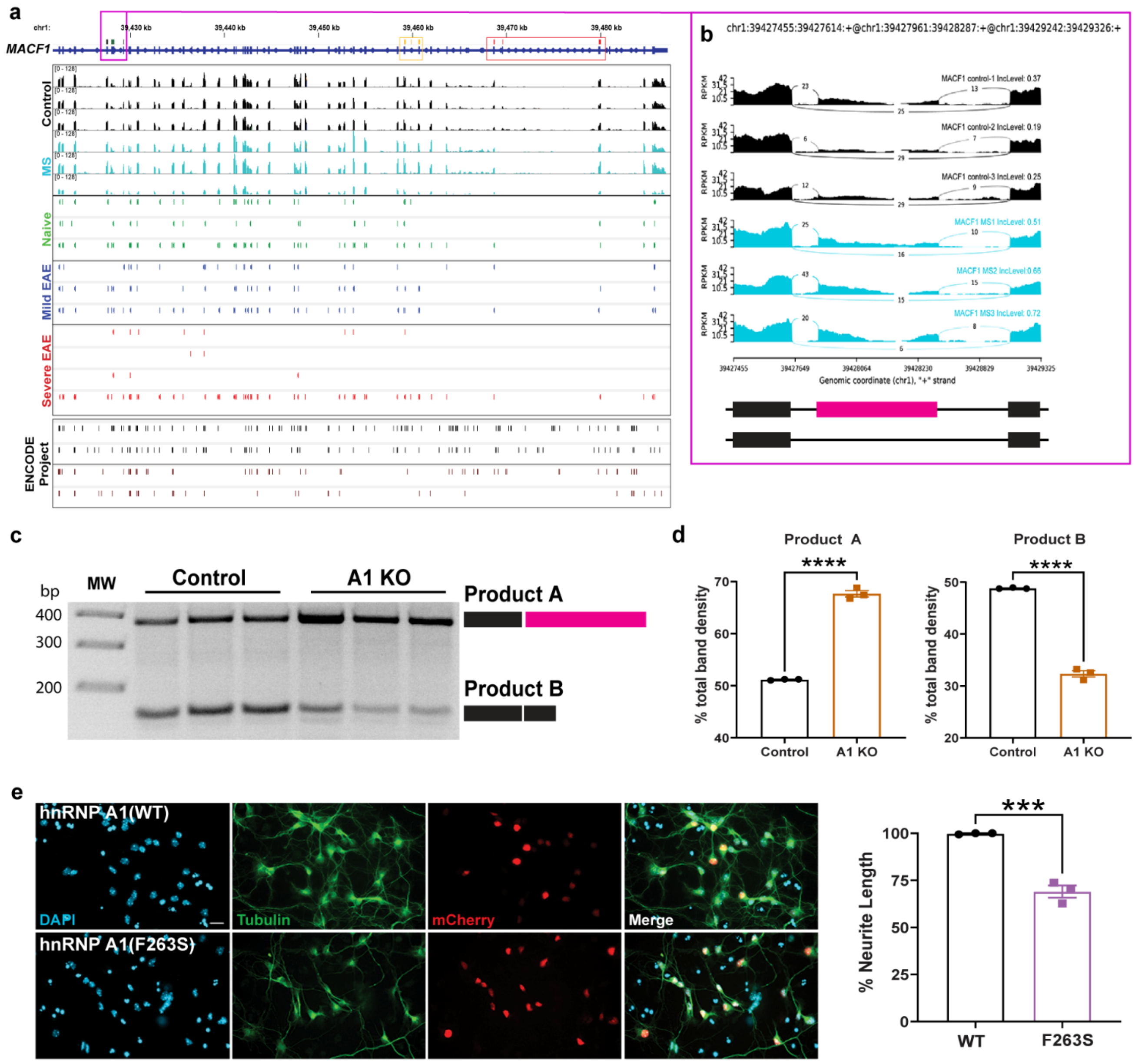

Supplementary Figure 9. hnRNP A1 dysfunction induces changes in *MACF1/Macf1* splicing resulting in neurite loss.
(a) RNA sequencing reads in *MACF1* from MS samples (light blue) as compared to controls (black) with rMATS identified alternative splicing events in three different areas of *MACF1* (magenta, yellow, red boxes). LiftOver was then used to overlap mouse CLIPseq tracks of hnRNP A1 binding sites on human *MACF1*. Data of hnRNP A1 binding sites on human *MACF1* from the ENCODE project were incorporated to confirm binding sites from previously published datasets. LiftOver of CLIPseq peaks demonstrates differential hnRNP A1 binding in EAE *vs*. naïve samples. (b) Sashimi plot of one of the areas of *MACF1* identified by rMATS (magenta box) demonstrates a significant change in exon usage. RNA sequencing reads from control (black) and MS cases (light blue) demonstrate increased reads within the middle exon (pink) in MS cases, illustrating it is more highly included in disease. (c, d) Products were analyzed by PCR to identify *Macf1* alternative splicing changes in Neuro2A cells with and without hnRNP A1 knockout (KO) (n=3 biological replicates for each) and detected by gel electrophoresis. Neuro2A cells with hnRNP A1 knockout show the same significant changes in exon usage within *Macf1* as EAE, MS, and primary neuron samples. Unpaired t-test, one-tailed with ****$p<0.0001$. Data are plotted as the mean +/- SEM. (e) Primary neurons transduced with hnRNP A1(F263S) (red) exhibit

decreased neurite length (tubulin, green) as compared to hnRNP A1(WT)-transduced neurons (n=3 biological replicates for WT and F263S). Scale bar 20 μm. Unpaired t-test, one-tailed with ***$p<0.001$ (exact $p=0.0003$). Data are plotted as mean +/- SEM.

Supplementary Table 4

Human sample demographics

Sample ID	Age	Sex	Clinical Diagnosis	Use
C1	82	M	N/A	RNA sequencing; PCR; WB
C2	40	F	N/A	RNA sequencing; PCR; WB
C3	62	M	N/A	RNA sequencing; PCR; WB
C4	34	M	N/A	PCR
C5	N/A	N/A	N/A	PCR
MS1	59	F	Progressive MS	RNA sequencing; PCR; WB
MS2	52	F	SPMS	RNA sequencing; PCR
MS3	31	F	PPMS	RNA sequencing; PCR; WB
MS4	46	M	SPMS	PCR
MS5	72	F	SPMS	PCR; WB
MS6	46	M	MS	WB
MS7	36	F	RRMS	WB
MS8	56	F	MS	WB

Male (M); Female (F); Multiple sclerosis (MS); Secondary progressive multiple sclerosis (SPMS); Primary progressive multiple sclerosis (PPMS); Relapsing remitting multiple sclerosis (RRMS); Western blot (WB)

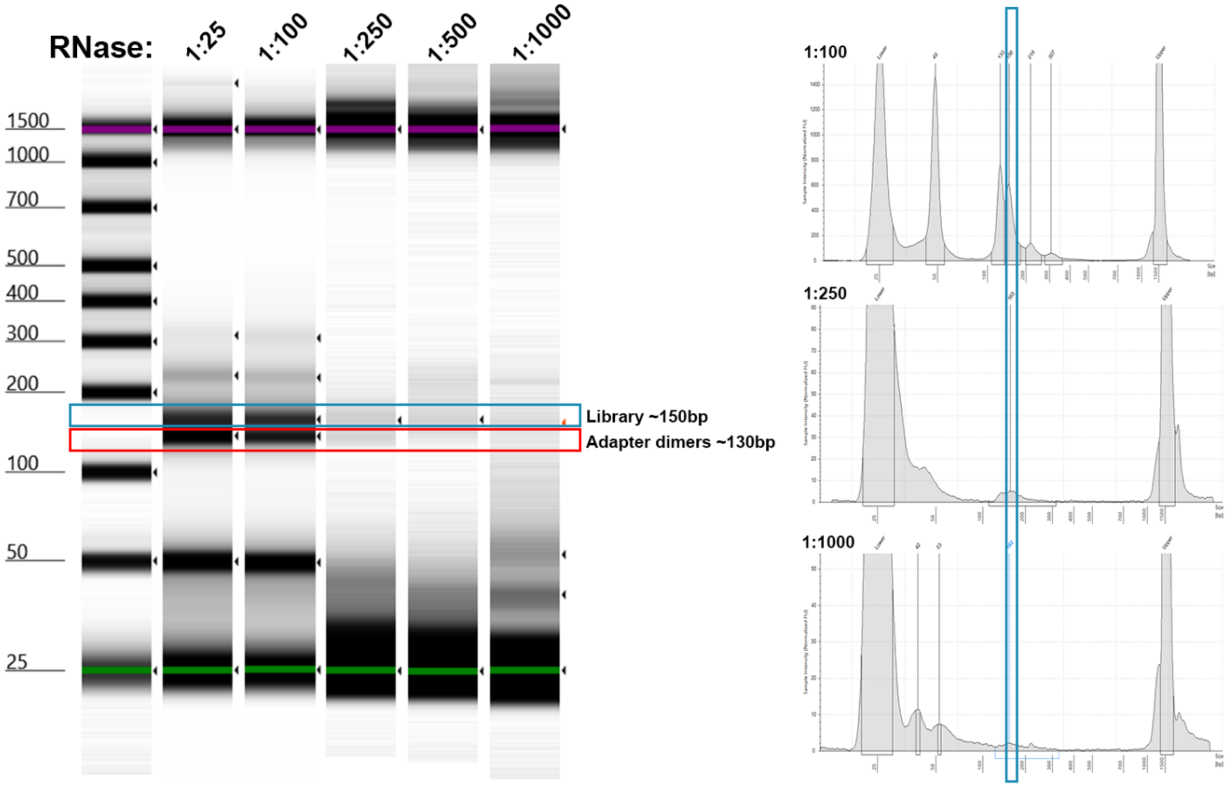

Supplementary Figure 10. Optimization of RNaseI concentrations.
Tapestation traces of RNaseI treatments (1:25, 1:100, 1:250, 1:500, 1:1000) to determine proper concentration for library construction via the NextFlex kit. The NextFlex kit adapter dimer products are approximately 130bp and indicate insufficient input RNA. A 1:250 dilution of RNaseI was the lowest dilution of RNase that yielded enough RNA to prevent adapter dimer accumulation and yielded peaks on the RNA Tapestation of approx. 156bp, without higher mass contaminants.

Supplementary Table 5

Primers for cloning, RT-PCR, amplicon PCR, and qPCR

Use	Species	Name	Sequence
Cloning	n/a	hSyn-Kpn-KOZAK-mCh-F	CTGAGAGCGCAGTCGAGAAGGATCCGGTACCGCCACCATGGTGAGCAAGGGCGAG
		AAV-HindIII-mCh-R	CCAGAGGTTGATTATCGATAAGCTTCTACTTGTACAGCTCGTCCATG
		hSyn-Kpn-A1-F	CTGAGAGCGCAGTCGAGAAGGATCCGGTACCGCCACCATGTCTAAGTCAGAGTCTCCTAAAGAGC
		ITR-qPCR-F	GGAACCCCTAGTGATGGAGTT
		ITR-qPCR-R	CGGCCTCAGTGAGCGA
		AAVS1-Puro-F	GGAGGAGAATCCCGGCCCTGCTAGCACCGAGTACAAGCCC
		AAVS1-Puro-R	GCTGATCAGCGAGCTCTAGGAATTCTCAGGCACCGGGCTTGC
		A1-gRNA03-P1	CACCGGGAACACTAACAGACTGTG
		A1-gRNA03-P2	AAACCACAGTCTGTTAGTGTTCCC
		T2A-NheI-Puro_F	GGAGGAGAATCCCGGCCCTGCTAGCACCGAGTACAAGCCC
		PolyA-EcoRI-Puro_R	GCTGATCAGCGAGCTCTAGGAATTCTCAGGCACCGGGCTTGC
		mhnRNPA1_gRNA-03-F	CACCGGGAACACTAACAGACTGTG
		mhnRNPA1_gRNA-03-R	AAACCACAGTCTGTTAGTGTTCCC
RT-PCR	Mouse	RT-mAbi2-R	GGCAGGCCTATTCATGCTG
		RT-mMacf1-i-R	GTCTTTGCTGAAACTGGGGTATTTGC
	Human	RT-hABI2-R	GGCAGGCCTATTCATGCTG
		RT-hMACF1-2078-R	GATCAGTGGTGGCATACGCAG
Amplicon PCR	Mouse	Ampl-mMacf1-i-Upstream-F	CTTGCTAACTCGGAACCTGTTGG
		Ampl-mMacf1-i-Middle-R	CTGCAGGACATCCTGTCTGTAATC
		Ampl-mMacf1-i-Downstream-R	CTGTAGTTTGTTTTAGAAGAGCCTGACC
		Ampl-mAbi2-Upstream-F	GTACCCAGAACATGAAGATGGG
		Ampl-mAbi2-Middle-R	GGTTGGGCTAGGTACGTAATC
		Ampl-mAbi2-Downstream-R	GACTGCTGCTCCGACTACTTG
		Ampl-mPkm-F	CTGAAGGCAGTGATGTGGCC
		Ampl-mPkm-R	CACGAAGGTCGACATCCTCAGC
	Human	Ampl-hMACF1-i-Upstream-F	GAGACAGCTGAGCCTATTTC
		Ampl-hMACF1-i-Middle-R	CAGAGCTTGGTCAAGTAGGG
		Ampl-hMACF1-i-Downstream-R	GGTTTGTTTTAGAAGAGCCTGAC
		Ampl-hPKM-F	CTGAAGGCAGTGATGTGGCC
		Ampl-hPKM-R	ACCCGGAGGTCCACGTCCTC
		Ampl-hABI2-Upstream-F	GTACCCAGAACATGAAGATGGG
		Ampl-hABI2-Middle-R	GGTTGGGCTAGGTACGTAATC
		Ampl-hABI2-Downstream-R	GACTGCTGCTCCGACTACTTG
qPCR	Mouse	qPCR-mActb_F	ACACCCGCCACCAGTTCG
		qPCR-mActb_R	CTTTGCACATGCCGGAGCC
		qPCR-mNrcam_F	CCGAAGCCGACTCTGGAAAT
		qPCR-mNrcam_R	TTTTGAGGTGCCACGATCCA
		qPCR-mTaf1_F	CATGGCCTACAATGTGCAGC
		qPCR-mTaf1_R	ATTGTCCTCCCAACGTCCAT
		qPCR-mErmn_F	CAGAAGACATGGCTTTCCGAG
		qPCR-mErmn_R	TGCTGGGCAGTTCTTTCCTTC
		qPCR-mFam107a_F	CGTGAGCTGCTTATGAACCAC
		qPCR-mFam107a_R	ATTCCTGCGACGGTGCTCTA
		qPCR-mHnrnpa2b1_F	GAAATCGGGCTGAAGCGACT
		qPCR-mHnrnpa2b1_R	AGGATCCCGCATAACCACAC
		qPCR-mSpock3_F	GAATGAGCAGTGCACCAAGG
		qPCR-mSpock3_R	TGTTACTGAGCTCCGTGTGG
		qPCR-mMapt_1F	CGCTGGGCATGTGACTCAA
		qPCR-mMapt_1R	TTTCTTCTCGTCATTTCCTGTCC
RNA-EMSA	Human	hMACF1 RNA	rGrGrCrUrCrUrGrGrArArGrArArGrArCrArUrArGrArArArArCrCrArUrGrCrArArCrArGrArUrGrUrGrCrArCrCrArGrGrCrArGrUmC

Supplementary Table 6

Plasmids used in this manuscript

Plasmid Name	Source	Reference
pAAV-hSyn-mScarlet	Addgene: 131001	Marshel, J. H., Kim, Y. S., Machado, T. A., Quirin, S., Benson, B., Kadmon, J., Raja, C., Chibukhchyan, A., Ramakrishnan, C., Inoue, M., Shane, J. C., McKnight, D. J., Yoshizawa, S., Kato, H. E., Ganguli, S., & Deisseroth, K. (2019). Cortical layer-specific critical dynamics triggering perception. Science, 365(6453). https://doi.org/10.1126/science.aaw5202
pAdΔF6	Addgene: 112867	pAdDeltaF6 was a gift from James M. Wilson (Addgene plasmid # 112867 ; http://n2t.net/addgene:112867 ; RRID:Addgene_112867)
pAAV2/9n	Addgene: 112865	pAAV2/9n was a gift from James M. Wilson (Addgene plasmid # 112865 ; http://n2t.net/addgene:112865 ; RRID:Addgene_112865)
N-pmCry2PHR-A1(WT)-mCherry-C	Levin Lab	Clarke, J.-P. W. E., Thibault, P. A., Salapa, H. E., Kim, D. E., Hutchinson, C., & Levin, M. C. (2021). Multiple Sclerosis-Associated hnRNPA1 Mutations Alter hnRNPA1 Dynamics and Influence Stress Granule Formation. International Journal of Molecular Sciences, 22(6), 2909. https://doi.org/10.3390/ijms22062909
pAAV-hSyn-mCherry	Levin Lab	This manuscript
pAAV-hSyn-hnRNPA1(WT)-mCherry	Levin Lab	This manuscript
pAAV-hSyn-hnRNPA1(F263S)-mCherry	Levin Lab	This manuscript
pU6-(BbsI)_CBh-Cas9-T2A-mCherry-H1-(BamHI)	Addgene: 64217	Chu, V. T., Weber, T., Wefers, B., Wurst, W., Sander, S., Rajewsky, K., & Kuhn, R. (2015). Increasing the efficiency of homology-directed repair for CRISPR-Cas9-induced precise gene editing in mammalian cells. Nature Biotechnology, 33(5). doi: 10.1038/nbt.3198
AAVS1_Puro_Tet3G_3xFLAG_Twin_Strep	Addgene: 92099	Dalvai, M., Loehr, J., Jacquet, K., Huard, C. C., Roques, C., Herst, P., Cote, J., & Doyon, Y. (2015). A scalable genome-editing-based approach for mapping multiprotein complexes in human cells. Cell Reports 13(3). doi: 10.1016/j.celrep.2015.09.009
pU6-(BbsI)_CBh-Cas9-T2A-PuroR-H1-(BamHI)	Levin Lab	This manuscript
pU6-(gA1-03)_CBh-Cas9-T2A-PuroR-H1-(BamHI)	Levin Lab	This manuscript
N-pmMBP-TEV-hnRNPA2-C	Levin Lab	Clarke JP, Thibault PA, Fatima S, Salapa HE, Kalyaanamoorthy S, Ganesan A, Levin MC. Sequence- and structure-specific RNA oligonucleotide binding attenuates heterogeneous nuclear ribonucleoprotein A1 dysfunction. Front Mol Biosci. 2023 Jun 22;10:1178439. doi: 10.3389/fmolb.2023.1178439. PMID: 37426420; PMCID: PMC10325567.
N-MBP-TEV-A1WT-C	Levin Lab	Clarke JP, Thibault PA, Fatima S, Salapa HE, Kalyaanamoorthy S, Ganesan A, Levin MC. Sequence- and structure-specific RNA oligonucleotide binding attenuates heterogeneous nuclear ribonucleoprotein A1 dysfunction. Front Mol Biosci. 2023 Jun 22;10:1178439. doi: 10.3389/fmolb.2023.1178439. PMID: 37426420; PMCID: PMC10325567.
N-MBP-C	Levin Lab	Clarke JP, Thibault PA, Fatima S, Salapa HE, Kalyaanamoorthy S, Ganesan A, Levin MC. Sequence- and structure-specific RNA oligonucleotide binding attenuates heterogeneous nuclear ribonucleoprotein A1 dysfunction. Front Mol Biosci. 2023 Jun 22;10:1178439. doi: 10.3389/fmolb.2023.1178439. PMID: 37426420; PMCID: PMC10325567.
