# Supplementary material for: hnRNP A1 dysfunction alters RNA splicing and drives neurodegeneration in multiple sclerosis (MS)
Source: Nat Commun. 2024 Jan 8;15:356. doi: 10.1038/s41467-023-44658-1 (PMC10774274; doi:10.1038/s41467-023-44658-1)
Supplement: Supplementary file 2 — Description of Additional Supplementary Files [file 41467_2023_44658_MOESM2_ESM.pdf]

Description of Additional Supplementary Files

File Name: Supplementary Data 1
Description: Human differentially expressed genes

File Name: Supplementary Data 2
Description: Gene ontology results for human differentially expressed genes

File Name: Supplementary Data 3
Description: Gene names of RNAs bound by hnRNP A1 in mouse spinal cords common between CLIPper and assembly approaches and used for subsequent analyses

File Name: Supplementary Data 4
Description: Gene ontology results for hnRNP A1-bound RNAs from mouse spinal cord
