# Supplementary material for: hnRNP A1 dysfunction alters RNA splicing and drives neurodegeneration in multiple sclerosis (MS)
Source: Nat Commun. 2024 Jan 8;15:356. doi: 10.1038/s41467-023-44658-1 (PMC10774274; doi:10.1038/s41467-023-44658-1)
Supplement: Supplementary file 4 — Reporting Summary [file 41467_2023_44658_MOESM4_ESM.pdf]

nature portfolio

Corresponding author(s): Dr. Michael C. Levin

Last updated by author(s): Dec 11, 2023

Reporting Summary

Nature Portfolio wishes to improve the reproducibility of the work that we publish. This form provides structure for consistency and transparency in reporting. For further information on Nature Portfolio policies, see our Editorial Policies and the Editorial Policy Checklist.

Statistics

For all statistical analyses, confirm that the following items are present in the figure legend, table legend, main text, or Methods section.

n/a	Confirmed	
☐	☒	The exact sample size (*n*) for each experimental group/condition, given as a discrete number and unit of measurement
☐	☒	A statement on whether measurements were taken from distinct samples or whether the same sample was measured repeatedly
☐	☒	The statistical test(s) used AND whether they are one- or two-sided<br>*Only common tests should be described solely by name; describe more complex techniques in the Methods section.*
☒	☐	A description of all covariates tested
☐	☒	A description of any assumptions or corrections, such as tests of normality and adjustment for multiple comparisons
☐	☒	A full description of the statistical parameters including central tendency (e.g. means) or other basic estimates (e.g. regression coefficient) AND variation (e.g. standard deviation) or associated estimates of uncertainty (e.g. confidence intervals)
☐	☒	For null hypothesis testing, the test statistic (e.g. F, t, r) with confidence intervals, effect sizes, degrees of freedom and P value noted<br>*Give P values as exact values whenever suitable.*
☒	☐	For Bayesian analysis, information on the choice of priors and Markov chain Monte Carlo settings
☒	☐	For hierarchical and complex designs, identification of the appropriate level for tests and full reporting of outcomes
☒	☐	Estimates of effect sizes (e.g. Cohen's *d*, Pearson's *r*), indicating how they were calculated

Our web collection on statistics for biologists contains articles on many of the points above.

Software and code

Policy information about availability of computer code

Data collection	Zeiss Zen 3.1 (blue edition); Fiji for ImageJ; QuantStudio Design & Analysis Software (v.1.5.1); GelCapture Imaging Software (Invitrogen); Olympus cellSens imaging software (v.1.4); Illumina NextSeq 550; Agilent TapeStation 4150 instrument; LI-COR Odyssey imaging system; Nanodrop 1000 spectrophotometer; Qubit 4.0 Fluorometer
Data analysis	Microsoft Excel for Microsoft Office; Prism 8 (GraphPad Software Inc); Cytoscape (v.3.8.2); Fiji for ImageJ (Neurite Tracer Analysis Suite); Fastp; rnaSPAdes v.3.15.3; minimap2 (v.2.17); bedtools (v.2.29.2); Image Lab software v.6.1 (Bio-Rad); QuantStudio Design & Analysis Software v.1.5.1; BioRender to generate summary figures; CLIPper; Galaxy (PEAKachu, MEME suite, RCAS); ENCODE eCLIP-seq Processing Pipeline v2.2; Python; HTSeq (v.0.11.3); rMATS (v.4.1.2); Image Studio Lite; bcl2fastq (v.2.19.0.316); NuDup (v.2.3); IGV (v.2.15.2); LiftOver (UCSC Genome Browser). Code for IDR was based on the ENCODE eCLIP-seq Processing Pipeline v.2.2 with minor modifications for CLIPseq data analysis. The implemented changes are available upon request.

For manuscripts utilizing custom algorithms or software that are central to the research but not yet described in published literature, software must be made available to editors and reviewers. We strongly encourage code deposition in a community repository (e.g. GitHub). See the Nature Portfolio guidelines for submitting code & software for further information.

Data

Policy information about availability of data

All manuscripts must include a data availability statement. This statement should provide the following information, where applicable:

- Accession codes, unique identifiers, or web links for publicly available datasets
- A description of any restrictions on data availability
- For clinical datasets or third party data, please ensure that the statement adheres to our policy

All data generated or analyzed during this study are included in this article and its Supplementary Information files. All requests for raw data and materials should be addressed to the corresponding author. Source data are provided with this paper. RNA sequencing data have been deposited in the Gene Expression Omnibus (GEO) database under the accession codes GSE207680 (human RNAseq) and GSE208093 (mouse CLIPseq) and will be available following publication of the manuscript.

Research involving human participants, their data, or biological material

Policy information about studies with human participants or human data. See also policy information about sex, gender (identity/presentation), and sexual orientation and race, ethnicity and racism.

Reporting on sex and gender	Tissue samples are from 13 unique patients (n=5 control and n=8 MS) with n=6 female for MS samples and n=1 female for control samples with information unable to be found on n=1 control case. Controls mean age=55 and MS mean age=50.
Reporting on race, ethnicity, or other socially relevant groupings	N/A
Population characteristics	See Supplementary Table 4
Recruitment	N/A
Ethics oversight	Study approval was granted by the University of Sasktachewan Biomedical Research Ethics Board (BIO#17-207).

Note that full information on the approval of the study protocol must also be provided in the manuscript.

Field-specific reporting

Please select the one below that is the best fit for your research. If you are not sure, read the appropriate sections before making your selection.

☒ Life sciences ☐ Behavioural & social sciences ☐ Ecological, evolutionary & environmental sciences

For a reference copy of the document with all sections, see nature.com/documents/nr-reporting-summary-flat.pdf

Life sciences study design

All studies must disclose on these points even when the disclosure is negative.

Sample size	Sample size was determined based on the number of fresh frozen human tissue samples with high quality RNA available. Sample size for mouse experiments was determined based on previous EAE experiments where n=3-5 is sufficient for examining clinical score differences between control and EAE given the variability of the EAE disease course. Given that naive animals demonstrate less variability, n=3 is sufficient. In our lab, these group numbers (n=3-5 for EAE and naive) are also sufficient to yield consistent immunohistopathological findings (PMID: 35618205, 36382566, 31755578). For in vitro experiments, replicate number (n=3) was determined based on previous studies in our lab using similar cell culture systems (i.e. same cell lines or data collection at experimental endpoint) due to low variability between replicates (PMID: 37426420, 34697074).
Data exclusions	Due to disparities in sex between control and MS samples because of tissue availability, known human sex associated genes, which could skew differential expression results, were removed from differential gene expression analysis.
Replication	Figure legends detail the number of biological or technical replicates for all experiments in this manuscript where at least 3 biological replicates per group were included.
Randomization	Mice were randomly assigned as naive or EAE prior to EAE induction. Primary neuron coverslips were randomly selected to be transduced with hnRNP A1(WT) or hnRNP A1 (F263S). Neuro2A cells were randomly selected to be transfected with single guide RNA targeting hnRNP A1. SK-N-SH cells were randomly selected to be treated with cycloheximide or DMSO. Randomization is not applicable to human brain samples as they are pre-determined to be either control and MS.
Blinding	For human experiments, researchers were blinded when quantifying hnRNP A1 mislocalization in samples and during library preparation. In order to determine differences between control and MS, sequencing samples were unblinded post-processing for differential gene expression. For mouse experiments, EAE mice were identified using colored tailed marks, a designation which was carried throughout clinical scoring and data analysis. During CLIPseq library preparation and sequencing, each mouse sample, including naive, was assigned a number. In

order to determine differences between naive and EAE, sequencing samples were unblinded post-processing. For primary neuron neurite quantification, images were blinded and both groups were processed simultaneously. Researchers were not blinded during amplicon PCR, any experimental system western blotting, or qPCR experiments.

Reporting for specific materials, systems and methods

We require information from authors about some types of materials, experimental systems and methods used in many studies. Here, indicate whether each material, system or method listed is relevant to your study. If you are not sure if a list item applies to your research, read the appropriate section before selecting a response.

Materials & experimental systems

n/a	Involved in the study
☐	☒ Antibodies
☐	☒ Eukaryotic cell lines
☒	☐ Palaeontology and archaeology
☐	☒ Animals and other organisms
☒	☐ Clinical data
☒	☐ Dual use research of concern
☒	☐ Plants

Methods

n/a	Involved in the study
☒	☐ ChIP-seq
☒	☐ Flow cytometry
☒	☐ MRI-based neuroimaging

Antibodies

Antibodies used

The following primary antibodies were used: mouse anti-hnRNP A1 (clone 4B10, Millipore 05-1521, 1:1000 for IHC, IF, ICC, and 1 ug/mg of lysate for IP); rabbit anti-NeuN (abcam ab177487, 1:1000); chicken anti-beta-tubulin 3 (Aves Labs TUJ, 1:500); mouse anti-beta-actin (clone 8H10D10, Cell Signaling Technology 3700S, 1:1000); rabbit anti-beta-actin (Cell Signaling Technology 4967S, 1:1000); rabbit anti-Abi2 (ProteinTech 14890-1-AP, 1:2000).
The following secondary antibodies were used: donkey anti-mouse Alexa Fluor 488 (Jackson Immunoresearch 715-546-151, 1:1000); donkey anti-rabbit Alexa Fluor 594 (Jackson Immunoresearch 711-586-152, 1:1000); biotin-conjugated anti-mouse IgG (Fisher Scientific Canada 45200581, 1:200); goat anti-mouse IgG-HRP-conjugate (Bio-Rad 1706516, 1:3000); goat anti-chicken IgY FITC (Aves Labs F-1005, 1:100); goat anti-rabbit IgG HRP-conjugate (Bio-Rad 1706515, 1:3000); IRDye 680LT donkey anti-mouse IgG (LI-COR 926-68022, 1:10,000)

Validation

All antibodies used were sourced from commercial vendors and were selected based on previous use and validation by our group and/or validation for use in human and mouse tissues for IHC, ICC, WB, IP, and IF applications.
Mouse anti-hnRNP A1 (clone 4B10, Millipore 05-1521; RRID AB_11213192): Antibody epitope validation by our group (PMID: 31872424) and extensive previous use by our group in ICC, IF, IHC, WB in mouse and human (PMID: 32608162, 30190085, 36382566, 31755578, 34697074).
Rabbit anti-NeuN (abcam ab177487; RRID AB_2532109): Antibody validated by company for IF in human with additional validation information on abcam website and use in >400 publications.
Chicken anti-beta-tubulin 3 (Aves Labs TUJ; RRID AB_2313564): Antibody validated by company for IF in mouse with additional validation information on company website and use in 20 publications.
Mouse anti-beta-actin (Cell Signaling Technology 3700S; RRID AB_2242334): Antibody validated by company for WB in mouse with additional validation information on company website and use in >2500 publications.
Rabbit anti-beta actin (Cell Signaling Technology 4967S; RRID AB_330288): Antibody validated by company for WB in human and mouse with additional validation information on company website and use in >3000 publications.
Rabbit anti-Abi2 (ProteinTech 14890-1-AP; RRID AB_2288997): Antibody test and validated by company for WB in mouse brain tissue with additional validation information on company website.
Donkey anti-mouse Alexa Fluor 488 (Jackson Immunoresearch 715-546-151; RRID AB_2340850): Antibody validated by company for recognition of mouse IgG with minimal cross reactivity for other species. Additional validation information on company website and use in >40 publications.
Donkey anti-rabbit Alexa Fluor 594 (Jackson Immunoresearch 711-586-152; RRID AB_2340622): Antibody validated by company for recognition of rabbit IgG with minimal cross reactivity for other species. Additional validation information on company website and use in >40 publications.
Biotin-conjugated anti-mouse IgG (Fisher Scientific 45200581; RRID AB_1062579): Antibody validated by company (Cytiva RPN1001) for recognition of mouse IgG with high species specificity and use in immunodetection.
Goat anti-mouse IgG-HRP conjugate (Bio-Rad 1706516; RRID AB_2921252): Blotting-grade double affinity purified with validation by company for use in western blotting applications.
Goat anti-chicken IgY FITC (Aves Labs F-1005; RRID AB_2313516): Antibody validated by company for recognition of chicken IgY with no cross reactivity for mammalian IgG.
Goat anti-rabbit IgG HRP-conjugate (Bio-Rad 1706515; RRID AB_11125142): Blotting-grade double affinity purified with validation by company for use in western blotting applications.
IRDye 680LT donkey anti-mouse IgG (LI-COR 926-68022; RRID AB_10715072): Affinity chromatography isolated antibody which reagents with heavy and light chains of mouse IgG. Company tested by ELISA to ensure minimal cross-reactivity with other species proteins. Antibody validated and tested for western blotting.

Eukaryotic cell lines

Policy information about cell lines and Sex and Gender in Research

Cell line source(s)	All cell lines (HEK293T, Neuro2A, SKNSH) were acquired from ATCC. Primary embryonic cortical mouse neurons were a mixed sex model in that male and female embryos (E14-16) were pooled before isolating neurons for culture.
Authentication	Cell line authenticating performed by the supplier (ATCC).
Mycoplasma contamination	Cell lines are routinely tested for mycoplasma contamination via fluorescence (DAPI or Hoescht) microscopy examination.
Commonly misidentified lines (See ICLAC register)	Cell lines were not listed in the ICLAC register.

Animals and other research organisms

Policy information about studies involving animals; ARRIVE guidelines recommended for reporting animal research, and Sex and Gender in Research

Laboratory animals	All animal experiments were conducted in approved University of Saskatchewan facilities using C57BL/6N mice (age 10 weeks for EAE studies and embryonic days 14-16 (E14-16) for primary embryonic neuron culture generation) purchased from Charles River Laboratories. Mice were housed five to a cage (for adult mice) or alone (pregnant dam for embryonic isolation) under pathogen-free conditions on a 12h/12h light:dark cycle with ad libitum access to standard rodent chow and water in 70-72F humidity-controlled rooms.
Wild animals	No wild animals were used in this study.
Reporting on sex	For EAE experiments, female mice were used as previously published by us. The active immunization protocol used by our group is recommended for use in females (Hooke Laboratories EK-2110) to induce more consistent disease. Primary neuron cultures were mixed sex in that cortices isolated from male and female embryos were pooled before isolating and culturing neurons.
Field-collected samples	No field-collected samples were used in this study.
Ethics oversight	All animal experiments were performed in accordance with the University of Saskatchewan's Animal Research Ethics Board under Animal Use Protocol (AUP) 20170104.

Note that full information on the approval of the study protocol must also be provided in the manuscript.

Plants

Seed stocks	Not applicable to this study.
Novel plant genotypes	Not applicable to this study.
Authentication	Not applicable to this study.
